# Supplementary material for: Mycobacterium tuberculosis Controls Phagosomal Acidification by Targeting CISH-Mediated Signaling
Source: Cell Rep. 2017 Sep 26;20(13):3188–98. doi: 10.1016/j.celrep.2017.08.101 (PMC5637157; doi:10.1016/j.celrep.2017.08.101)
Supplement: Document S1. Supplemental Experimental Procedures and Figures S1–S7 [file mmc1.pdf]

Cell Reports, Volume *20*

Supplemental Information

Mycobacterium tuberculosis Controls Phagosomal Acidification by Targeting CISH-Mediated Signaling

Christophe J. Queval, Ok-Ryul Song, Jean-Philippe Carralot, Jean-Michel Saliou, Antonino Bongiovanni, Gaspard Deloison, Nathalie Deboosère, Samuel Jouny, Raffaella Iantomasi, Vincent Delorme, Anne-Sophie Debrie, Sei-Jin Park, Joana Costa Gouveia, Stanislas Tomavo, Roland Brosch, Akihiko Yoshimura, Edouard Yeramian, and Priscille Brodin

Supplemental Information

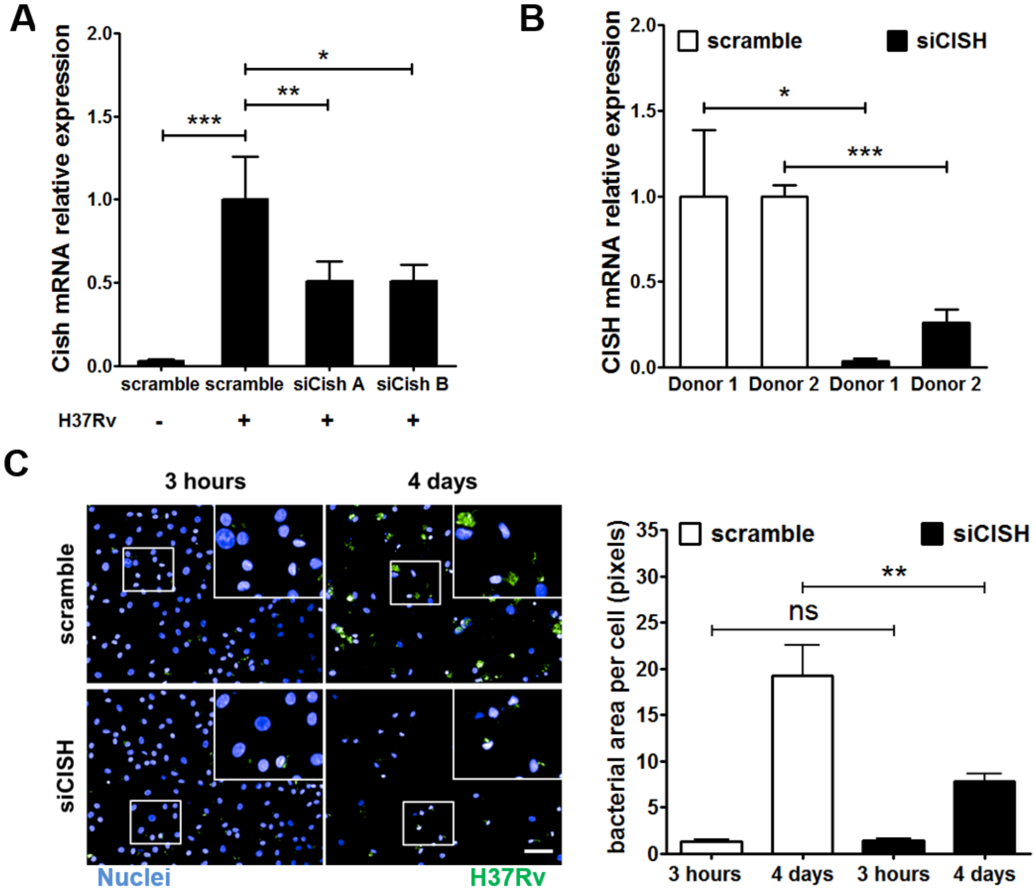

Supplemental Figure S1, Related to Figure 1. Inhibition of *Mtb* intracellular replication by CISH silencing

(**A**) Cish mRNA expression by RT-qPCR in siCishA, siCishB or scramble RAW 264.7 cells upon infection with H37Rv at 3 h p.i. (**B**) CISH mRNA relative expression quantified by RT-qPCR in siCISH and scramble hMΦ upon infection. The data reported correspond to the CISH mRNA expression ±SD obtained for two independent donors (black bars: donor 1; grey bar: donor 2) (**C**) Typical confocal images of siCISH and scramble hMΦ upon infection with H37Rv-GFP. Image based quantification of the infection 4 days p.i. For all data, statistical significances were determined using student t-test. * $p<0.05$, ** $p<0.01$, *** $p<0.001$, NS: not significant.

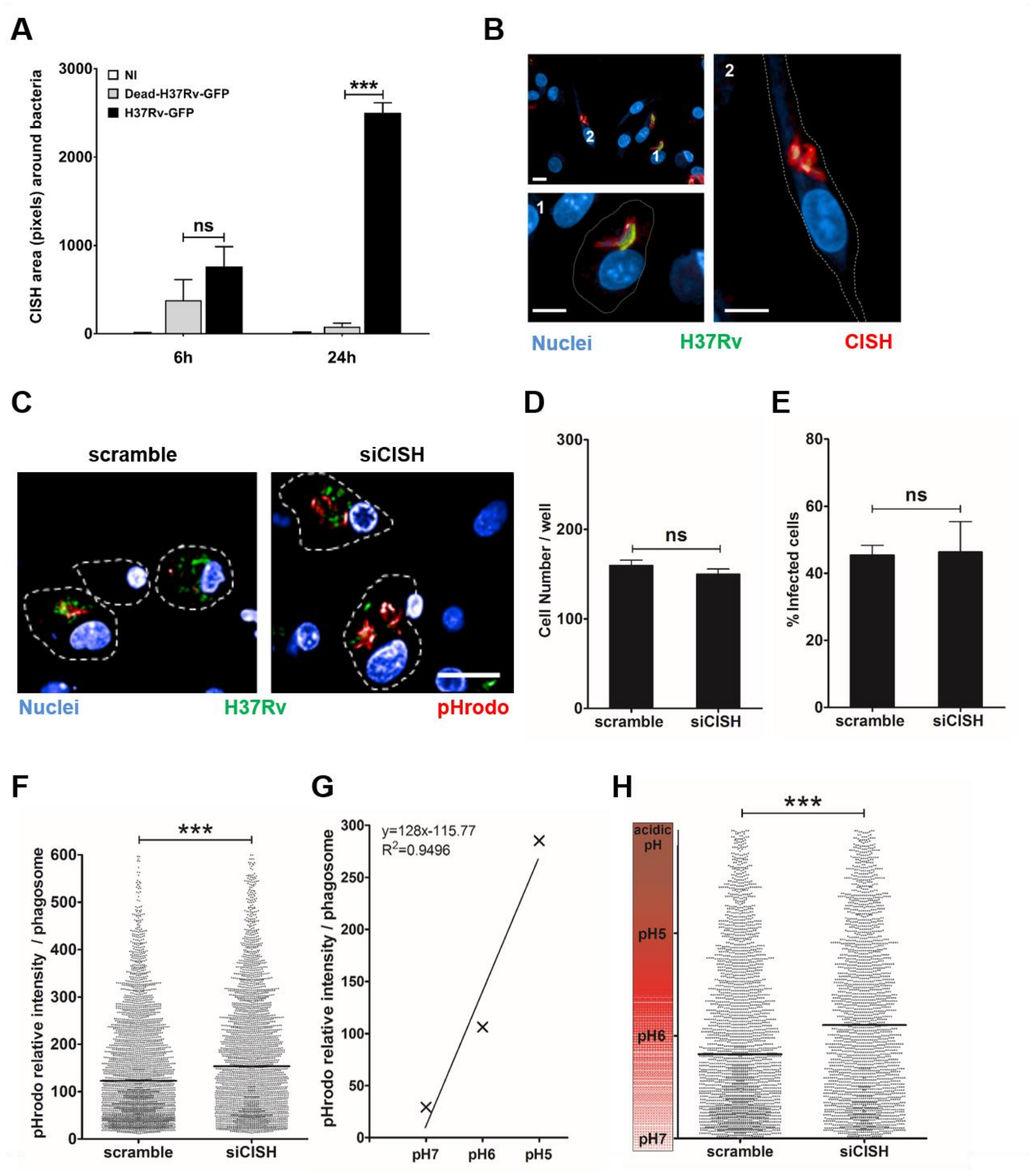

Supplemental Figure S2, Related to Figure 2. Vacuolar recruitment of CISH and phagosomal acidification upon *Mtb* infection in human primary macrophages (**A**) Analysis of the recruitment of Cish around Mtb H37Rv and Formalin-killed H37Rv-GFP (Dead-H37Rv-GFP) fluorescence microscopy in RAW 264.7 cells. Plot represents the recruitment of CISH (area) detected around dead or live H37Rv-GFP bacteria. *** p

<0.001, ns: non-significant. (**B**) Intracellular localization of CISH, in hMΦ infected with H37Rv-GFP for 6 h, was visualized using fluorescence microscopy. Nuclei were labelled with DAPI (blue), H37Rv-GFP bacteria were visualized in green and CISH was immunolabelled using CISH antibody (red). Scale bar, 10 µm. During the image acquisition an intensity cut-off was applied to avoid fluorescence saturation, which implies that lower concentrations of CISH localized elsewhere in the cells might not be visible in these images (**C**-**H**) Comparison of *Mtb*-containing phagome acidification in hMΦ, control (scramble) or silenced for CISH (siCISH), and infected at MOI 2 for 24 h with H37Rv-GFP labelled with pHrodo dye. (**C**) Typical confocal microscope images of hMΦ infected with pHrodo-labelled H37Rv-GFP Scale bar, 20 µm. (**D** and **E**) Image-based quantification of cell number (**D**) and percentage of infected cells (**E**). (**F**) Mean pHrodo relative intensity per phagosome. Plotted values are for siCISH (N phagosomes = 2860) and scramble controls (N phagosomes = 2920). Black bars represent the medians. (**G**) Standard curve representing the pHrodo relative intensity of bacteria in function of pH (**H**) Estimation of phagosomal pH obtained by converting pHrodo relative intensity / phagosome (**F**) in pH value according to the standard curve. Black bars represent the medians. All data are representative of 2 independent experiments, each performed with 4 replicates. ns: no-significant, ***p<0.001 (Mann-Whitney test).

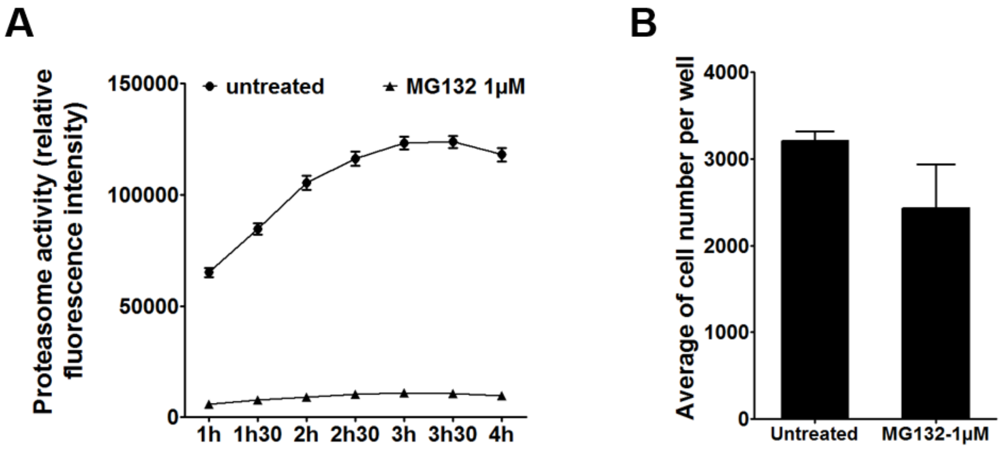

Supplemental Figure S3, Related to Figure 3. Monitoring proteasome activity in *Mtb*-infected macrophages (A) Kinetics of proteasome 20S activity in hMΦ treated with proteasome inhibitor MG132 (1 µM) or not (Untreated). **(B)** Control of cell viability by assessment of the number of cells in presence of MG132.

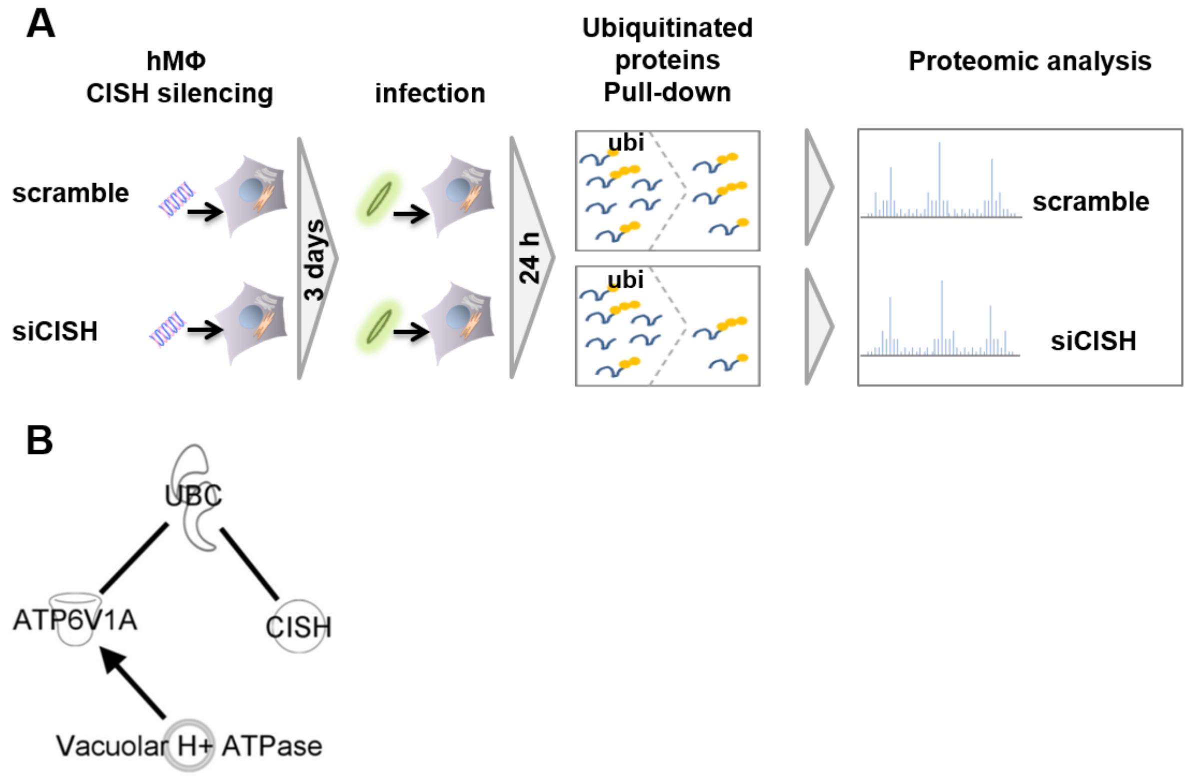

C

		Number of peptides			Number of spectra		
Protein name	accession number	siCISH	scramble	ratio	siCISH	scramble	ratio
ATP6V1A	P38606	6 ±1.4	10 ±1.4	0.6	7.5 ±0.7	15 ±1.4	0.5

Supplemental Figure S4, Related to Figure 4. High throughput proteomic analysis for the identification of V-ATPase subunit A as a target of CISH for ubiquitylation (**A**) Pull-down of ubiquitylated-proteins and their analysis by high throughput proteomics of extracts of siCISH and scramble hMΦ after *Mtb* H37Rv-GFP infection. (**B**) Scheme displaying the interactions between CISH, the ubiquitin system (UBC) and VATPase subunit A (ATP6V1A). (**C**) Number of peptides and spectra detected by proteomics for the identification of ATP6V1A as a potential CISH target for ubiquitination. Values represent the mean±SD of the peptides and spectra detected in *Mtb*-infected siCISH or scramble hMφ from two donors. The complete data are presented in Table S1.

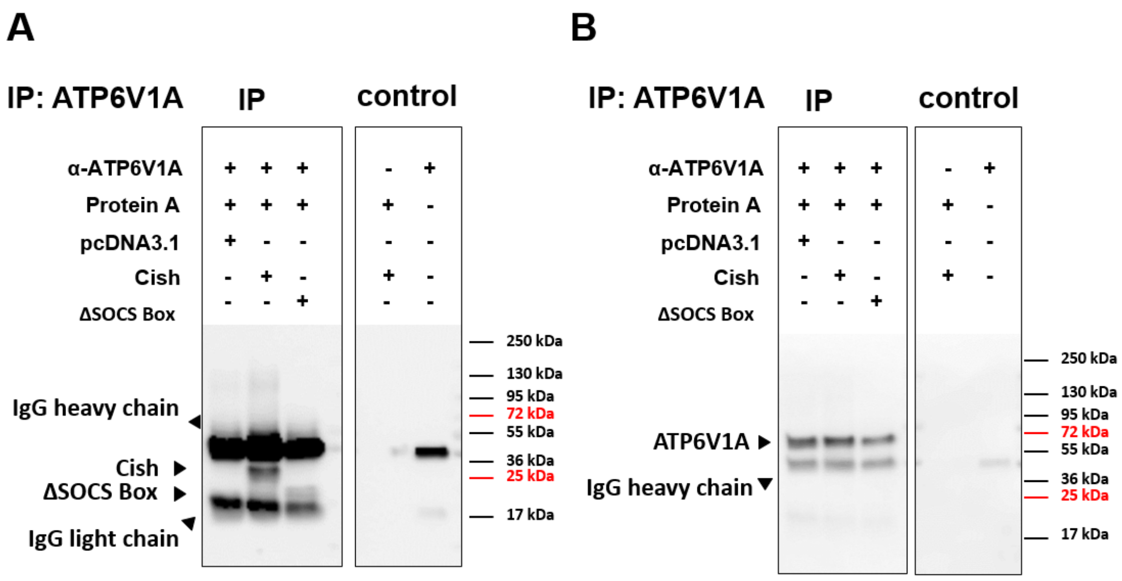

Supplemental Figure S5, Related to Figure 4. Cish interacts with V-ATPase subunit A (**A** and **B**) Whole immunoblot obtained after immunoprecipitation of endogenous ATP6V1A in HEK 293 cells transfected with pcDNA 3.1-Cish, pcDNA 3.1-CishΔSOCS Box or control empty vector pcDNA 3.1. (**A**) Immunoblotting of Cish showing the presence of Cish in cells transfected with pcDNA 3.1-Cish with contamination of immunoglobulin used for immunoprecipitation (heavy and light chain). (**B**) Membrane was then stripped and probed using an anti-ATP6V1A.

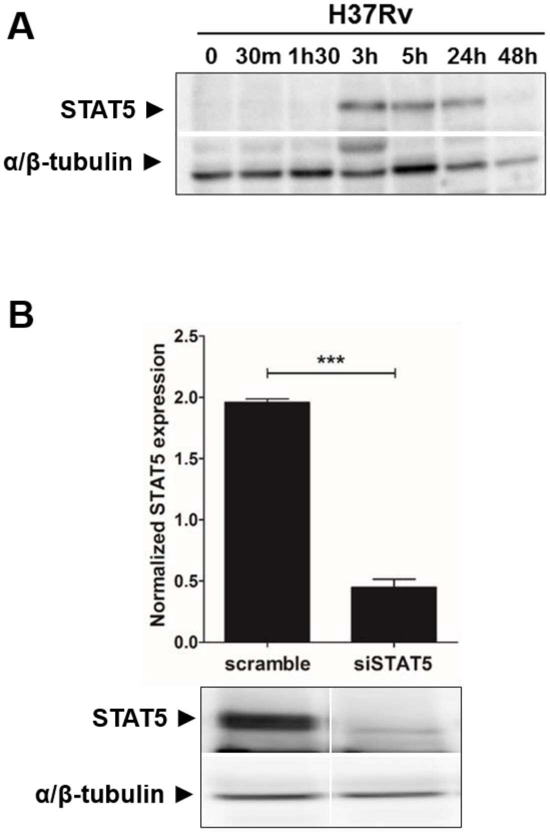

Supplemental Figure S6, Related to Figure 5. STAT5 activation in *Mtb*-infected macrophages is required for CISH expression (**A**) Kinetics of STAT5 activation in RAW 264.7 macrophages infected with H37Rv. STAT5 activation was analyzed by immunoblotting using anti-PY^{694}-STAT5. Blotting with anti- αβ -tubulin was used to confirm gel loading. Immunoblots are representative of two independent experiments. (**B)** siSTAT5 and scramble hMΦ were infected with H37Rv-GFP for 4 h. The graphic represents the quantification of STAT5 relative phosphorylation. Reported values represent the means ±SEM of three independent experiments. For all data, statistical significances were determined using student t-test. * $p<0.05$, ** $p<0.01$, *** $p<0.001$, ns: not significant.

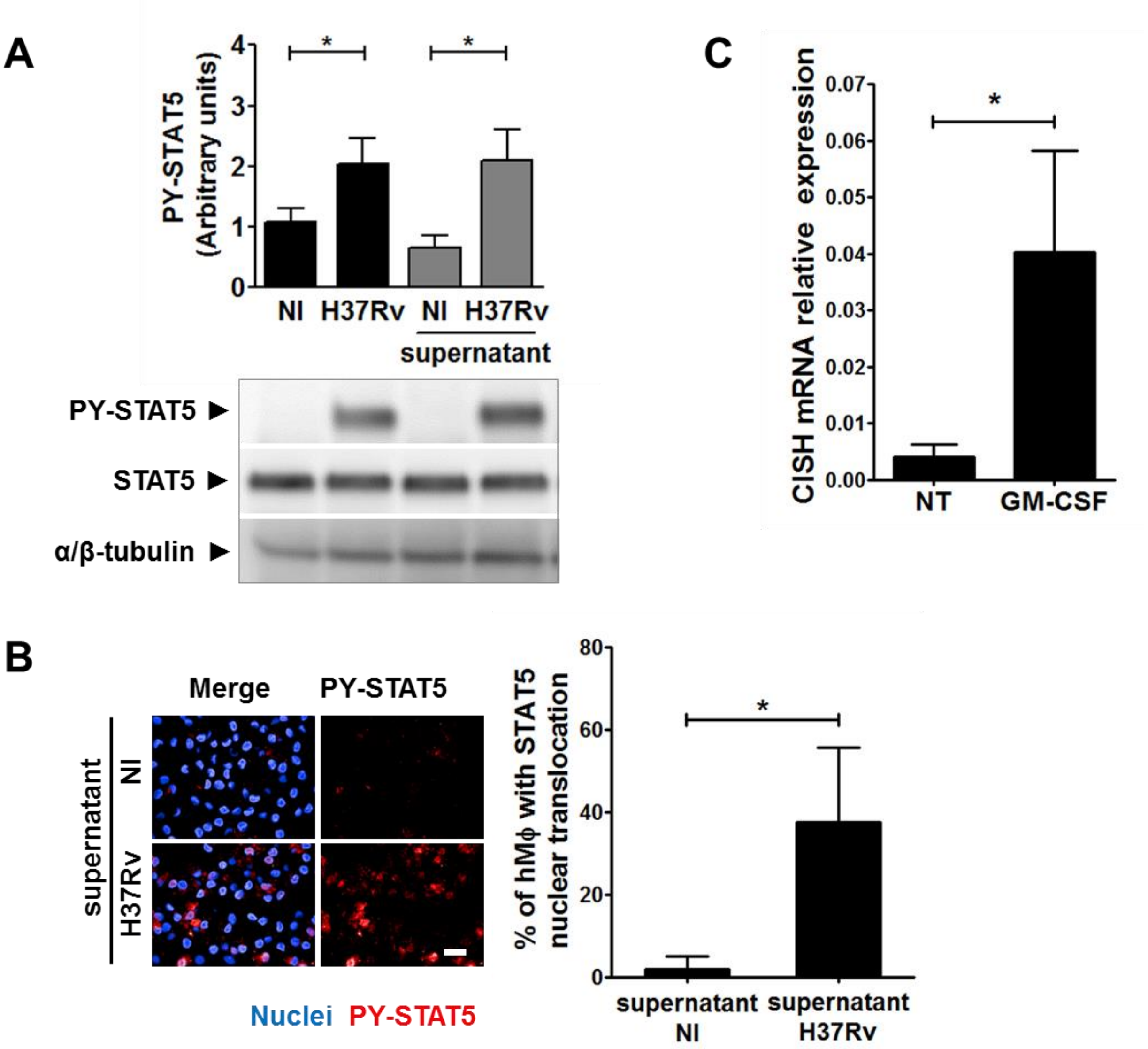

Supplemental Figure S7, Related to Figure 6. STAT5-mediated CISH expression depends on GM-CSF release by *Mtb*-infected macrophages. (**A**) STAT5 activation was analyzed by immunoblotting using anti-PY^{694}-STAT5 in hMΦ that had been infected with *Mtb* (H37Rv, black box) or not (NI, black box) or incubated with cell culture supernatant from hMΦ (H37Rv, grey box) and non-infected hMΦ (NI, grey box). Probing with anti-STAT5 and anti-α/β-tubulin antibodies was used to confirm gel loading. (**B**) Confocal images of hMΦ that were incubated for 3 h with filtered supernatant from uninfected hMΦ (NI) or supernatant from infected hMΦ (H37Rv). Macrophage nuclei were stained using DAPI (blue) and phosphorylated form STAT5 were detected using anti-PY^{694}-STAT5 (red). Micrographs are representative of four independent

experiments with four different donors. Scale bar, 20 µm. The graphic represents the mean percentage of hMΦ presenting STAT5 nuclear translocation. (**C**) Monitoring of CISH mRNA expression by RT-qPCR in hMΦ that were treated with 50 ng/mL of human GM-CSF for 3 h. Non-treated cells (NT) were used as negative control. The data correspond to the means ±SD of relative CISH mRNA expression from 2 independent experiments. For all data, statistical significances were determined using student t-test. * $p<0.05$, ** $p<0.01$, *** $p<0.001$, ns: not significant.

Supplemental Experimental Procedures

Antibodies and reagents

Polyclonal rabbit anti-PY^{694}-STAT5 (#9359), anti-STAT5 (#9363), anti-CISH (#8731) and anti-α/β-tubulin (#2148) were purchased from Cell Signaling Technology. Mouse anti-Poly-Ub (FK1/#BML-PW8805) was from ENZO. Rabbit Anti-ATP6V1A antibody (#17115-1-AP) was purchased from ProteinTech. For western blotting, the antibodies were probed using secondary donkey anti-rabbit antibodies, conjugated with horseradish peroxidase (Jackson Immunoresearch). For immunofluorescence, the antibodies were probed using secondary donkey anti-rabbit antibodies conjugated to Rhodamine Red X (Jackson Immunoresearch). Cell nuclei were fluorescently labelled using DAPI (Sigma-Aldrich) or SYTO60 (Life Technologies). Purified human GM-CSF and pure grade neutralizing anti-GM-CSF antibody were purchased from Miltenyi Biotech.

SiRNA individual duplexes

Non-targeting siRNA (scramble, target sequence: 5'-U AAG GCU AUG AAG AGA UAC-3') from Dharmacon Thermofisher (Boulder, CO, USA), siCishA (5'-CAGTTATACAGTATTTATTTA-3'), siCishB (5'-AAAGCAAGTGTTAGAACACAA-3') were resuspended at 2 µM in QIAGEN resuspension buffer, aliquoted and stored at -20°C until use.

Mammalian cells

RAW 264.7 cells (ATCC TIB-71) were grown to 60-80% confluence in RPMI 1640 medium (Difco) supplemented with 10% heat-inactivated FBS (Life Technologies). $CD14^+$ hMΦs were isolated from human peripheral blood monocytes (hPBMCs) as previously described (Queval et al., 2014). Briefly, hPBMCs were purified by Ficoll density gradient centrifugation and isolated using a $CD14^+$ magnetic bead purification kit (Miltenyi Biotech). Purified $CD14^+$ monocytes were then differentiated into macrophages by 6-day incubation in RPMI 1640 complemented with 10% FBS and 40 ng/mL colony stimulating factor (hM-CSF) (Miltenyi Biotech) at 37°C in 5% CO_2. For all secondary cell lines used in this manuscript, the maximum number of passage after thawing was 10.

Mycoplasma test was routinely performed and all secondary cell lines used were tested negative for mycoplasma.

Mycobacterial strains and cell infection

A recombinant strain of *Mtb* H37Rv expressing the enhanced green fluorescent protein (H37Rv-GFP) was cultured in Middlebrook 7H9 medium (Difco) supplemented with 10% oleic acid-albumin-dextrose-catalase (OADC, Difco), 0.2% glycerol, 0.05% Tween 80 (all from Sigma-Aldrich, St. Louis, MO, USA), and 50 µg/ml hygromycin (Invitrogen) until the exponential phase was reached (Christophe et al., 2009). Bacilli were washed with Dulbecco's Phosphate Buffered Saline (DPBS free from $MgCl_2$ and $CaCl_2$, Gibco) resuspended in 10 mL of RPMI 1640 medium containing 10% FBS and decanted for 1 h at room temperature to allow bacterial aggregates to sediment. Bacterial titre was determined by measuring EGFP fluorescence on a Victor Multilabel Counter (Perkin Elmer). The bacterial suspension was diluted at the required titre in RPMI 1640 supplemented with 10% FBS prior to infection.

***In vivo* experiments**

6 week-old female C57BL/6 Cish knock-out mice (previously described elsewhere(Matsumoto et al., 1999)) and wild-type mice (ORIENTBIO Inc., SouthKorea) were challenged with *Mtb* H37Rv via the intranasal route with 10 µl of a suspension containing 5.10^5 organisms/ml. The day of infection (day 0 (n=4), 21 (n=4) and 42 days (n=4), lungs were homogenized by use of an MM300 apparatus (Qiagen) and 2.5-mm diameter glass beads. Serial 10-fold dilutions in medium were plated on 7H11 agar and colony forming unit counts were ascertained at 37°C after 3 weeks of growth. CFUs are reported for 4 mice per group per time point. Each cage hosted different groups. The experimentator had access to the label of the cages and, for each group, took mice randomly in the cages for each time point.

CFU determination

Cells were washed before lysis at the indicated times after bacterial infection. Serial dilutions were performed in DPBS and three drops of each dilution were plated onto

7H11 agar plates supplemented with 10% OADC. *Mtb* colony numbers were determined and the average of three spots of at least two dilutions were considered to determine the intracellular CFU per well. The CFUs were normalized to the total cell numbers (CFU/well) of the corresponding wells, using data from image analysis.

Cell-based siRNA assays

RAW 264.7 cells were reverse transfected with 100 nM of siRNA, in duplicate, using 0.2% Lipofectamine LTX (Invitrogen) in 384-well tissue culture plates (Greiner, Frickenhausen, Germany). Non-targeting siRNA (scramble) from Dharmacon Thermofisher (Boulder, CO, USA) was used to transfect cells in 48 wells per 384-well assay plate. Three days after transfection, one replicate of transfected cells was infected with H37Rv-GFP at a multiplicity of infection (MOI) of 3. Infection was allowed to proceed for 5 h at 37°C and 5% CO_2, and extracellular bacilli were removed via extensive washing of the plate, five times with 60 µl of DPBS-1% FBS using the Aquamax DW4 plate washer from Molecular Devices (Sunnyvale, CA, USA). Antibiotics were never used at any of the multiple steps of the experiments. Infected RAW 264.7 cells were resuspended in 50 µl of RPMI with 10% FBS and incubated at 37°C and 5% CO_2 for 5 days. As a positive control, 32 wells per 384-well plate were treated with 5 µg/ml of isoniazid (INH) from Sigma-Aldrich. On day 5 after infection, cells were stained with 10 µM of SYTO60 far red fluorescent nucleic acid stain (Invitrogen) and confocal images were acquired using an automated fluorescence microscope Opera (Perkin Elmer) equipped with a 20×/0.7 NA water immersion lens, driven by a robotic platform from EVOTEC AG (Hamburg, Germany). Of the four available light sources, 488- and 635-nm confocal excitation lines were used in order to excite GFP and SYTO60 red, respectively. Emission was detected simultaneously using two CCD cameras in spectral bands at 535 and 690 nm. Three fields for each well were acquired. Images from 384-well plates were analyzed as described previously (Christophe et al., 2009). To detect the intracellular bacteria, each field was analyzed using image-based analysis software Acapella 2.6 (Perkin Elmer). For each field, two channels were recorded: one for the green fluorescent-bacteria (green) and one for the cell nuclei (blue channel). 2-color images were then segmented using the following implementation of procedures: *i*) nuclei

detection was performed using a built-in Acapella procedure, *ii*) cytoplasm detection, based on the nuclei population, was performed using a built-in Acapella procedure, *iii*) bacteria detection was performed by retaining only pixels whose intensities were higher than a manually-defined threshold, *iv*) to identify infected cells the positions of cells and those of bacteria were merged together. Final results, expressed as the averages of the corresponding fields, concerned, respectively, the total bacterial area, the total number of cells, the percentage of infected cells and the bacterial area per cell (average of all infected cells).

SiRNA transfection of human primary macrophages

SiRNA transfection of primary hMΦs was adapted from the protocol previously described (Troegeler et al., 2014). Briefly, 5 days after differentiation with human M-CSF (40 ng/mL), primary CD14$^+$ hMΦs were collected for reverse transfection using the HiPerFect transfection reagent from Qiagen. Silencing was performed with siRNA from Dharmacon. For STAT5 silencing, 50 nM of pooled STAT5A siRNA (D-005169-01, D-005169-02, D-005169-05, D-005169-06) and pooled STAT5B siRNA (D-010539-01, D-010539-03, D-010539-05, D-010539-18) was used. For CISH silencing, 50 nM of pooled CISH siRNA (D-017381-01, D-017381-02, D-017381-03, D-017381-04) was used. Of note, in mice only one variant of Cish mRNA is expressed (NM_009895.3), whereas in humans 2 variants of CISH mRNAs are found (CISH variant 1: NM_013324.5; CISH variant 2: NM_145071.2). These pooled CISH siRNA target the two variants. As a control, macrophages were transfected with 50 nM of pooled Non-targeting siRNA (scramble) (D-001810-10). Transfected cells were then incubated for 3 days in RPMI 1640 supplemented with 10% FBS and 20 ng/mL M-CSF at 37°C, 5% CO_2. Transfected cells were then infected with *Mtb,* and samples were collected at the indicated time points.

Detection of CISH mRNA by RT-qPCR

After infection, macrophages were washed once with DPBS and mRNA was purified by using the RNeasy purification kit from QIAGEN, according to the manufacturer's recommendations. For each sample tested, 1 µg of mRNA was then reverse-transcribed

using the Reverse Transcription System kit from PROMEGA. Relative expression of *CISH* cDNA was detected using specific FAM-labeled Taqman Probe from Life Technologies which targeted mouse *Cish* (Mm01230623) or human *CISH* variants 1 and 2 (Hs00367082). We used *GAPDH* as a housekeeping gene. GAPDH was detected using FAM-labeled Taqman Probe from Life technologies targeting mouse *Gapdh* (Mm99999915) or human *GAPDH* (Hs02758991). FAM fluorescence was detected using LightCycler 480 (ROCHE), and the crossing points (Cp) of PCR reactions were calculated using the Basic Relative Quantification method. Data were then normalized and quantified using the $2^{-\Delta Cp}$ method.

Electrophoresis and immunoblotting

Cells were washed with DPBS (Difco), lysed using 62.5 mM Tris-HCl (pH 6.8), 10% glycerol, 4% SDS, 0.025% bromophenol blue and 1.5% β-mercaptoethanol, and boiled at 100°C for 15 min. The proteins were resolved by SDS-PAGE in precast acrylamide gels 4%-15% (BioRad) and transferred onto a polyvinylidene difluoride (PVDF) membrane. The PVDF membranes were blocked in DPBS, 0.1% Tween 20, 5% BSA and probed overnight with the appropriate antibodies (CISH 1/100, PY-STAT5 1/1000). Blots were then incubated with horseradish peroxidase-conjugated secondary antibodies (Jackson Immunoresearch), followed by detection using chemiluminescence, according to the manufacturer’s instructions (Immobilon, Millipore). The chemiluminescent signal was detected using the LAS 3000 Pro Bio Imaging Analyser. The fluorescence intensity of each spot was quantified using the ImageJ software.

Immunofluorescence for image based-quantification of CISH, poly-Ub, V-ATPase subunit A and PY-STAT5

After infection, hMΦs were fixed with 10% neutral buffered Formalin solution (HT5014, Sigma-Aldrich) for 30 min and permeabilized with cold methanol for 10 min at -20°C for STAT5 detection or with PBS-Triton X-100 0.2% for 4 min at RT for CISH, ubiquitin and V-ATPase subunit A detection. Cells were then incubated with blocking buffer (DPBS, 1% FBS) for 30 min prior to overnight incubation at 4°C with rabbit anti-CISH (1/100), rabbit anti-Poly-Ub (1/100) and rabbit anti-PY^{694}-STAT5 (1/100) antibodies. Cells were

then washed 3 times with DPBS and incubated with donkey anti-rabbit conjugated with Rhodamine Red X for 1 h at room temperature. Cells were washed again 3 times with DPBS and incubated for 10 min with 2.5 µg/mL DAPI in DPBS. The buffer was replaced by D-PBS containing 1% FBS. Plates were sealed and stored at 4°C. Confocal images were acquired on an automated fluorescent confocal microscope Opera (Perkin Elmer) using a 20X or 60X water lens (NA 0.7), 561 nm laser and 600 nm camera. 8 fields per well were recorded on average. Each image was processed using Columbus image analysis software (Perkin Elmer). For the quantification of the percentage of colocalization between *Mtb* H37Rv and CISH (Fig. 2C), GFP-bacteria spots were first detected using a built-in Acapella procedure as described elsewhere(Queval et al., 2016). The size of each spot was extended by 3 pixels on each direction leading to "the bacterial ring region". CISH fluorescence area was detected in the red channel by retaining only pixels which intensity was higher than a manually-defined threshold. The ratio of the "bacterial ring region" that was positive for CISH was then quantified. For the quantification of STAT nuclear translocations, PY-STAT5 staining was segmented using built-in scripts, and the ratio of cells positive for PY-STAT5 was determined for each well (Fig. 5)(Queval et al., 2016).

For the quantification of CISH/V-ATPase colocalization around *Mtb* vacuole, confocal images were acquired using a confocal microscope Zeiss LSM880 equipped with a Plan-Apochromat 63x/1,4 NA lens and laser 405, 488, 561, 633 nm. During the image acquisition an intensity cut-off was applied to avoid fluorescence saturation, which implies that lower concentrations of CISH localized elsewhere in the cells might not be visible in these images. Fluorescence intensities from *Mtb*-GFP, CISH and V-ATPase subunit A were analyzed using ImageJ and the plugin JACoP (Bolte and Cordelieres, 2006). The colocalization between CISH and V-ATPase subunit A was determined by the calculation of the Pearson's correlation coefficient in the area that encompasses the bacteria by an extra 1 µm (Bolte and Cordelieres, 2006).

Quantification of phagosomal acidification by LysoTracker

3000 RAW 264.7 cells were reverse transfected in 384-well plates (Carralot et al., 2009) with 100 nM of non-targeting (scramble) siRNA or with CISH-targeting siRNA duplexes

A & B using 0.2% Lipofectamine LTX (Invitrogen), and further incubated at 37°C and 5% CO_2 for four days. As a positive control, three days after transfection, cells were activated with recombinant mouse Interferon-gamma (IFNγ, R&D Systems) and Lipopolysaccharide (LPS, Sigma) at 0.1 Units/ml and 10 ng/ml, respectively. Four days after transfection, cells were infected with *Mtb* H37Rv at an MOI of 10:1. Infection was allowed to proceed for 2 h at 37°C and 5% CO_2, and extracellular bacilli were extensively washed out 5 times with 60 µl of DPBS-1% FBS. Infected RAW 264.7 cells were labelled with 1 µM of LysoTracker red DND-99 (Invitrogen) and 10 µg/ml of Hoechst 33342 (Sigma-Aldrich) for 1 h at 37°C and 5 % CO_2. Cells were washed four times with 60 µl of DPBS-1% FBS then fixed with 40 µl of 10% formalin solution for 10 min. Cells were subsequently washed twice with 60 µl of DPBS-1% FBS and stored in 1% FCS in PBS at 4°C until image acquisition. Confocal images were acquired using an Opera automated fluorescence microscope equipped with a 40×/0.7 NA water immersion lens. 405-, 488-, and 561-nm confocal excitation lines were used in order to excite Hoechst 33342, EGFP and LysoTracker red, respectively. Emission was detected simultaneously using two CCD cameras in spectral bands at 450, 520 and 600 nm. Four fields for each well were measured. Image analysis for the quantification of "the total acidic compartment surface proximal to cell nuclei" was performed using an algorithm described previously and Columbus software (Brodin et al., 2010).

Quantification of phagosomal acidification by pHrodo

Prior to cell infection, a two weeks old Mtb H37Rv-GFP culture was washed twice with DPBS buffer and then was incubated for 1 h at 37°C in 100 mM sodium bicarbonate, pH 8.5, containing 0.5 mM of amine-reactive pHrodo Red succinimidyl ester (Lifetechnologies). Labelled-bacteria were washed 3 times by centrifugation in DPBS buffer. hMΦs were infected with pHrodo-labelled and GFP-expressing Mtb at a MOI of 2. 24 h after infection, macrophage nuclei were labelled with Hoechst 33342 and confocal images were acquired on live cells using an OPERA automated fluorescence microscope equipped with a 60X water immersion lens (NA 0.7). 405-, 488-, and 561-nm confocal excitation lines were used in order to excite Hoechst 33342, EGFP and pHrodo red, respectively. Emission was detected simultaneously using two CCD cameras in

spectral bands at 450, 520 and 600 nm. For the quantification of pHrodo intensity around phagosomes, GFP-bacteria spots were first detected using a built-in Acapella procedure as described elsewhere (Queval et al., 2016). The size of each spot was extended by 4 pixels leading to “bacterial phagosome region”. For each phagosome, pHrodo fluorescence intensity was detected in the red channel and data were plotted as mean relative pHrodo intensity per phagosome.

Determination of the standard curve pHrodo relative intensity / bacteria = f(pH):

Mtb H37rv-GFP labelled with pHrodo were incubated in CO_2 independent medium (Gibco) at various pH (pH10 to pH4). The stained bacteria were then imaged using automated confocal microscopy and analysed using the same parameters as those used in samples. pHrodo mean intensity was measured for each bacterium detected (at least N=100) and the intensity was plotted as a median pHrodo intensity per bacterium (as shown in Supplementary Fig. S2F).

Quantification of proteasome activity

hMΦs transfected with siCISH or scramble were plated in a 384-well microplate and infected for 24 h with H37Rv-DsRed. As control, scramble cells were treated for 4 h with proteasome inhibitor MG132 1µM. Proteasome activity was quantified using Amplite^{TM} Fluorimetric Proteasome 20S Activity Assay Kit from AAT Bioquest. Cells were incubated 3 h with Proteasome LLVY-R110 substrate and Assay buffer according to the manufacturer’s recommendations and the fluorescence was collected using a plate reader Victor X3 (PerkinElmer), with filters Ex/Em = 490/525 nm.

Pull-down of ubiquitinated-proteins

hMΦs were first transfected with siCISH or scramble prior to infection with H37Rv-GFP at MOI of 1. 24 h post-infection, cells were washed once with PBS and lysed using buffer containing 20mM Tris HCl pH 7.5, 140mM NaCl, 1mM EDTA, 2% CHAPS (3-[((3-cholamidopropyl)-dimethylammonio]-1-propanesulfonate)), 10% glycerol, 10mM N-Ethylmaleimide. Cells were then collected and incubated in lysis buffer for 1 h at 4°C. Ubiquitinated-proteins were then pulled-down using UbiQapture kit from Enzolifesciences. For each sample, 700 µg of cell lysate was incubated with 80 µL of

UbiQapture-Q Matrix for 4.5 h at 4°C. UbiQapture-Q Matrix was then washed 5 times with PBS. Ubiquitinated-proteins that were bound to UbiQapture-Q Matrix were then eluted by boiling at 95°C for 15 min in 150µL PBS-containing SDS-PAGE buffer (62.5 mM Tris HCl pH 6.8, 4% SDS). UbiQapture-Q Matrix was then removed by short centrifugation. Eluted fractions that contain Ubiquitinated-proteins were collected into microtubes and stored at -20°C until analysis.

Proteomic analysis

After denaturation in a buffer 10 mM Tris-HCl pH 8 containing 5% SDS, 5% β-mercaptoethanol, 1 mM EDTA and 10% glycerol for 3 min, protein samples were fractionated on 10% acrylamide SDS-PAGE gel. The electrophoretic migration was stopped when the protein sample migrated at 1-cm into the separating gel. The gel was briefly stained with Coomassie Blue, and five bands containing the whole sample were cut. In-gel digestion of gel slices was performed as previously described (Miguet et al., 2009). An UltiMate 3000 RSLC nano System (Thermo Fisher Scientific) was used for separation of the protein digests. Peptides were automatically fractionated onto a C18 reversed phase column (75 µm×150 mm, 2 µm particle, PepMap100 RSLC column, Thermo Fisher Scientific, temperature 35 °C). Trapping was performed during 4 min at 5 µL/min with solvent A (98 % H2O, 2% ACN and 0.1 % FA). Elution was carried out using two solvents A (0,1 % FA in water) and B (0,1 % FA in ACN) at a flow rate of 300 nL/min. Gradient separation was 3 min at 5% B, 37 min from 5 % B to 30% B, 5 min to 80% B, and maintained at 80% for 5 min. The column was equilibrated for 10 min with 5% buffer B prior to the next sample analysis. The eluted peptides from the C18 column were analyzed by Q-Exactive instruments (Thermo Fisher Scientific). The electrospray voltage was 1.9 kV, and the capillary temperature was 275 °C. Full MS scans were acquired in the Orbitrap mass analyzer over m/z 300–1200 range with resolution 35,000 (m/z 200). The target value was 5.00E+05. Ten most intense peaks with charge state between 2 and 4 were fragmented in the HCD collision cell with normalized collision energy of 27%, and tandem mass spectrum was acquired in the Orbitrap mass analyzer with resolution 17,500 at m/z 200. The target value was 1.00E+05. The ion selection threshold was 5.0E+04 counts, and the maximum allowed ion accumulation times were

250 ms for full MS scans and 100 ms for tandem mass spectrum. Dynamic exclusion was set to 30 s.

Proteomic data analysis

Raw data collected during nanoLC-MS/MS analyses were processed and converted into *.mgf peak list format with Proteome Discoverer 1.4 (Thermo Fisher Scientific). MS/MS data were interpreted using search engine Mascot (version 2.4.0, Matrix Science, London, UK) installed on a local server. Searches were performed with a tolerance on mass measurement of 0.2 Da for precursor and 0.2 Da for fragment ions, against a composite target-decoy database (40488 total entries) built with Human Swiss-Prot database (TaxID=9606, 20 January 2015, 20198 entries) fused with the sequences of recombinant trypsin and a list of classical contaminants (46 entries). Cysteine carbamidomethylation, methionine oxidation, protein N-terminal acetylation, cysteine propionamidation and lysine ubiquitination (Gly-Gly) were searched as variable modifications. Up to three trypsin-missed cleavages were allowed. For each sample, peptides were filtered out according to the cut-off set for protein hits with 1 or more peptides longer than 7 residues, ion score > 30, identity score > 0 and a false positive identification rate less of than 0.5%.

We then selected the most representative proteins by removing all proteins displaying a mean number of peptides <3 in scramble control macrophages, in two independent biological replicates. The proteins targeted by CISH for ubiquitination were then determined by comparison of the relative number of proteins detected in siCISH macrophages to that of scramble control. The list of proteins presented in Table S1 was obtained according to the following criteria: i) ratio (number of peptides siCISH/number of peptides scramble) < 0.6; ii) ratio (number of spectra siCISH/number of spectra scramble) < 0.6; iii) number of peptides scramble - number of peptides siCISH ≥3.

Immunoprecipitation of V-ATPase subunit A

To immununoprecipitate endogenous V-ATPase catalytic subunit A (ATP6V1A), hMΦs were transfected with siCISH or scramble prior to infection with H37Rv-GFP for 24 h. To block protein degradations by proteasome, infected cells were treated with proteasome

inhibitor MG132 1µM 5 h. Cells were lysed in CHAPS buffer as described above (Pull-down of ubiquitinated-proteins, Materials and Methods). For each sample, 1 mg of cell lysate was incubated for 5 h at 4°C with 30 µL of Protein A Sepharose CL-4B beads (GE Healthcare) in presence of rabbit anti-ATP6V1A antibody (Proteintech Europe). The immune complexes were successively washed twice with buffer 1 (20mM Tris HCl, pH 7.5, 140mM NaCl, 1mM EDTA, 0.5% CHAPS, 10% Glycerol, 10mM N-Ethylmaleimide) and further washed 3 times with buffer 2 (20mM Tris HCl, pH 7.5, 280mM NaCl, 1mM EDTA, 10% Glycerol, 10mM N-Ethylmaleimide). The immune complexes were finally boiled for 15 minutes in loading buffer (62.5 mM Tris, pH 6.8, 20% Glycerol, 1.5% SDS, 0.025% bromophenol blue, 8% β-mercaptoethanol) before analysis by SDS-PAGE. After electrophoresis and transfer into PVDF membrane, ATP6V1A was immunoblotted using rabbit anti-ATP6V1A (Novus) and secondary antibody easy blot anti-rabbit IgG conjugated with HRP (GeneTex). Ubiquitin was revealed using Ubiquitin-conjugated specific HRP-linked antibody (Enzo Life Sciences).

Transfection of HEK 293 cells with pcDNA 3.1 encoding for Cish

HEK 293 cells (from ATCC) were grown to 80% confluence in RPMI 1640 medium (Difco) supplemented with 10% heat-inactivated FBS (Life Technologies). One day before plasmid transfection, 10^6 HEK 293 cells were harvested in a T-25 flask with 8 mL of complete growth medium. For each condition 8 µg of control pcDNA 3.1 or pcDNA 3.1-Cish were diluted in PBS to a final volume of 375 µL and mixed with 25 µL of FuGENE HD reagent (Promega). Transfectant-DNA complexes were then incubated 10 min at room temperature before incubation with the cells for 48 h at 37°C in an atmosphere containing 5% CO_2.

Human 30-Plex cytokine assay

Primary hMΦs were infected with *Mtb* H37Rv at a MOI of 1.for 5 or 24 h, cell culture supernatants were then filtered using a 0.22 µM PVDF filter, sampled and stored at -80°C until analysis. GM-CSF release was quantified using Human cytokine Magnetic 30-plex kit (Life Technologies) according to the manufacturer's protocol.

Supernatant transfer assay

hMΦs were first infected with *Mtb* H37Rv at a multiplicity of infection (MOI) of 2 for 3 h, then the supernatant was collected and filtered using a 0.22 µm PVDF filter to remove any residual bacteria. Non-infected hMΦs were further incubated with filtered supernatant for 3 h. Cells were collected, and STAT5 activation was determined by western blotting.

Neutralization of GM-CSF activity

Primary CD14$^+$ hMΦs were infected for 3 h with *Mtb* H37Rv at a MOI of 2, in presence of 10 µg/mL of neutralizing anti-GM-CSF (α-GM-CSF) antibody or control IgG. Phosphorylation of STAT5 was analyzed by electrophoresis and immunoblotting as described above.

Statistical analysis

In this work statistical analyses were performed using Student's t-test, with the exception of Figures 3B and 3F, for which Wilcoxon Mann-Whitney test was used.

Ethics statement.

Animal studies were carried out in strict accordance with the recommendations from the Animal Protection Law in Korea. The protocol IPK-10008 entitled New Target for anti-TB drug development was approved by the Institutional Animal Care and Use Committee of Institut Pasteur Korea. All efforts were made to minimize suffering of the animals. Each group (WT and Cish KO) was hosted in different cages. Human monocytes were purified from blood samples obtained from healthy blood adult donors under strict anonymity (Etablissement Français du Sang “Nord de France”, EFS, Lille). Written informed consents were obtained from the donors under EFS contract n°NT/18/2016/200 with respect to Decree n°2007-1220 (articles L1243-4, R1243-61 and following) dated August 10th 2007 of the French Public Health Code (published in the Official Journal of the French Republic of August 14th 2007). The use of human samples was approved by the French Ministry of Education and Research under the agreement DC 2015-2575.

References (Supplemental dataset)

Bolte, S., and Cordelieres, F.P. (2006). A guided tour into subcellular colocalization analysis in light microscopy. Journal of microscopy *224*, 213-232.

Brodin, P., Poquet, Y., Levillain, F., Peguillet, I., Larrouy-Maumus, G., Gilleron, M., Ewann, F., Christophe, T., Fenistein, D., Jang, J.*, et al.* (2010). High content phenotypic cell-based visual screen identifies Mycobacterium tuberculosis acyltrehalose-containing glycolipids involved in phagosome remodeling. PLoS pathogens *6*, e1001100.

Carralot, J.P., Kim, T.K., Lenseigne, B., Boese, A.S., Sommer, P., Genovesio, A., and Brodin, P. (2009). Automated high-throughput siRNA transfection in raw 264.7 macrophages: a case study for optimization procedure. Journal of biomolecular screening *14*, 151-160.

Christophe, T., Jackson, M., Jeon, H.K., Fenistein, D., Contreras-Dominguez, M., Kim, J., Genovesio, A., Carralot, J.P., Ewann, F., Kim, E.H.*, et al.* (2009). High content screening identifies decaprenyl-phosphoribose 2' epimerase as a target for intracellular antimycobacterial inhibitors. PLoS pathogens *5*, e1000645.

Matsumoto, A., Seki, Y., Kubo, M., Ohtsuka, S., Suzuki, A., Hayashi, I., Tsuji, K., Nakahata, T., Okabe, M., Yamada, S.*, et al.* (1999). Suppression of STAT5 functions in liver, mammary glands, and T cells in cytokine-inducible SH2-containing protein 1 transgenic mice. Molecular and cellular biology *19*, 6396-6407.

Miguet, L., Bechade, G., Fornecker, L., Zink, E., Felden, C., Gervais, C., Herbrecht, R., Van Dorsselaer, A., Mauvieux, L., and Sanglier-Cianferani, S. (2009). Proteomic analysis of malignant B-cell derived microparticles reveals CD148 as a potentially useful antigenic biomarker for mantle cell lymphoma diagnosis. Journal of proteome research *8*, 3346-3354.

Queval, C.J., Song, O.R., Deboosere, N., Delorme, V., Debrie, A.S., Iantomasi, R., Veyron-Churlet, R., Jouny, S., Redhage, K., Deloison, G.*, et al.* (2016). STAT3 Represses Nitric Oxide Synthesis in Human Macrophages upon Mycobacterium tuberculosis Infection. Scientific reports *6*, 29297.

Queval, C.J., Song, O.R., Delorme, V., Iantomasi, R., Veyron-Churlet, R., Deboosere, N., Landry, V., Baulard, A., and Brodin, P. (2014). A microscopic phenotypic assay for the quantification of intracellular mycobacteria adapted for high-throughput/high-content screening. Journal of visualized experiments : JoVE, e51114.

Troegeler, A., Lastrucci, C., Duval, C., Tanne, A., Cougoule, C., Maridonneau-Parini, I., Neyrolles, O., and Lugo-Villarino, G. (2014). An efficient siRNA-mediated gene silencing in primary human monocytes, dendritic cells and macrophages. Immunology and cell biology.
