# Supplementary figures and images for: Mycobacterium tuberculosis Controls Phagosomal Acidification by Targeting CISH-Mediated Signaling
Source: Cell Rep. 2017 Sep 26;20(13):3188–98. doi: 10.1016/j.celrep.2017.08.101 (PMC5637157; doi:10.1016/j.celrep.2017.08.101)

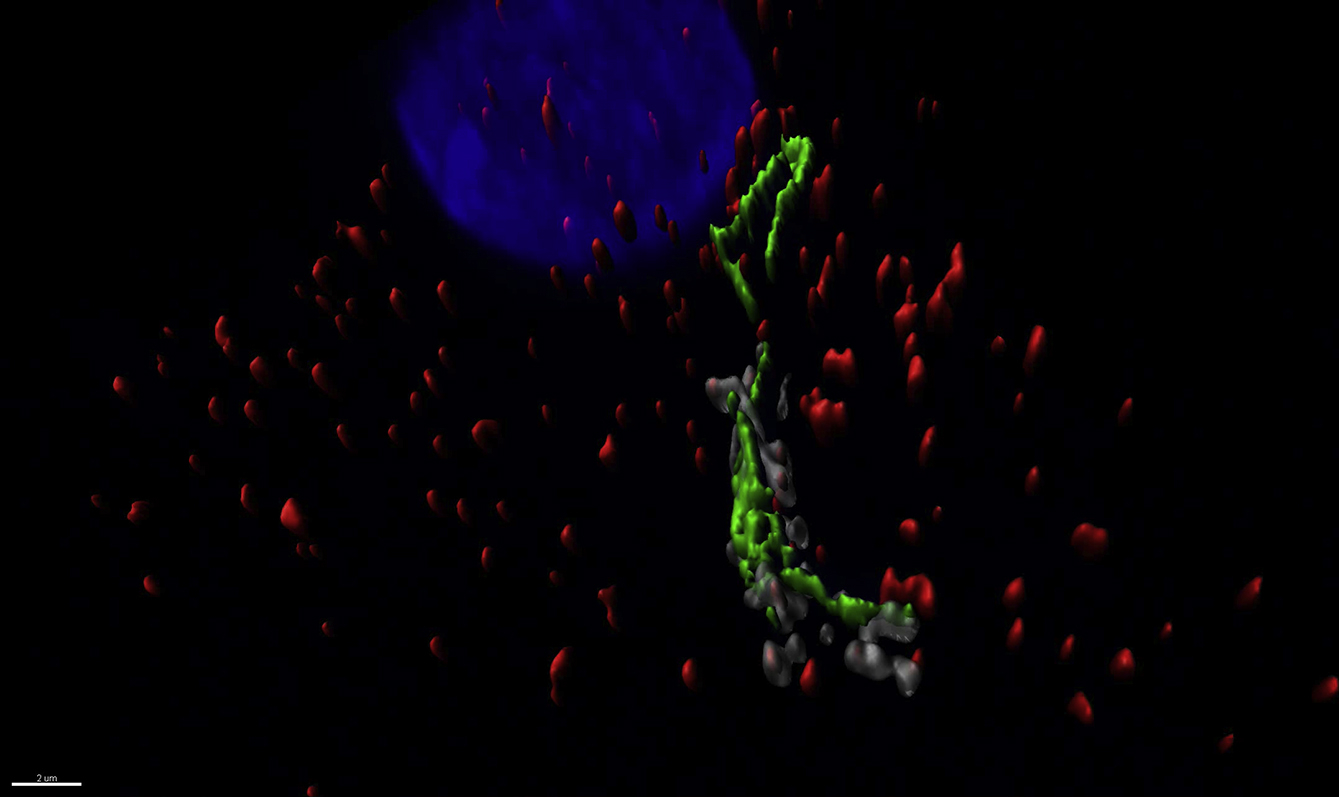

Supplement: Movie S1. Intracellular Localization of CISH and V-ATPase Subunit A Analyzed by Fluorescence Confocal Microscopy in Human Macrophages, Related to Figure 3E — DAPI-labelled nucleus is shown in blue, H37Rv-GFP bacteria are shown in green, CISH in grey and V-ATPase subunit A in red. [file mmc3.jpg]
