# Supplementary material for: Mycobacterium tuberculosis Controls Phagosomal Acidification by Targeting CISH-Mediated Signaling
Source: Cell Rep. 2017 Sep 26;20(13):3188–98. doi: 10.1016/j.celrep.2017.08.101 (PMC5637157; doi:10.1016/j.celrep.2017.08.101)
Supplement: Document S2. Article plus Supplemental Information [file mmc4.pdf]

Cell Reports

Article

Mycobacterium tuberculosis Controls Phagosomal Acidification by Targeting CISH-Mediated Signaling

Graphical Abstract

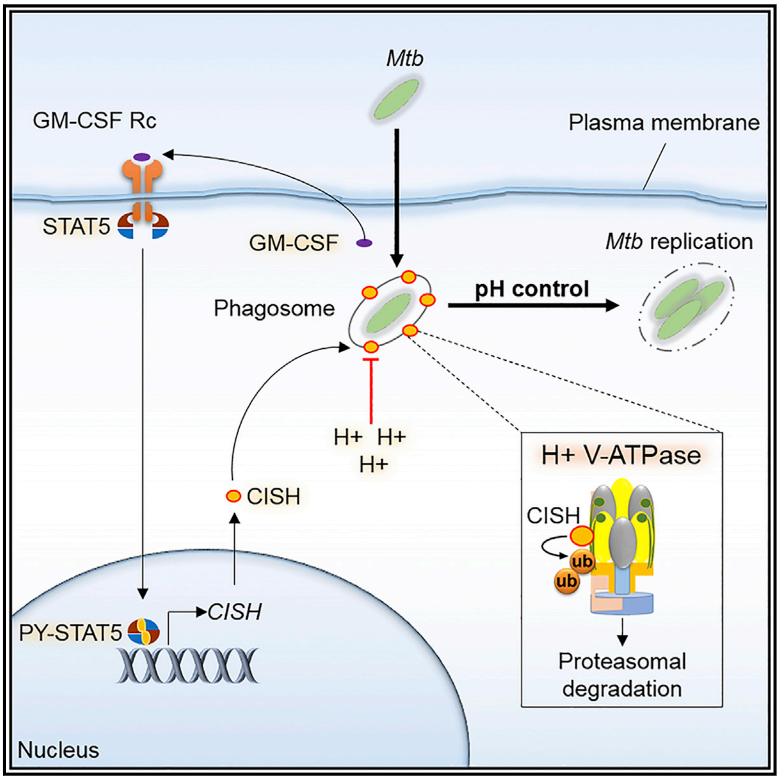

Highlights

- *M. tuberculosis* interferes with host pathways to control vacuolar acidification
- Infection induces the expression of host CISH and recruitment to the phagosome
- CISH triggers the degradation of H^{+}-V-ATPase via SOCS box-mediated ubiquitination
- This defense mechanism complements previous schemes relying on virulence factors

Authors

Christophe J. Queval, Ok-Ryul Song, Jean-Philippe Carralot, ..., Akihiko Yoshimura, Edouard Yeramian, Priscille Brodin

Correspondence

edouard.yeramian@pasteur.fr (E.Y.), priscille.brodin@inserm.fr (P.B.)

In Brief

Mycobacterium tuberculosis is effective at controlling phagosomal acidification. Queval et al. unravel a cellular mechanism underlying this defense strategy and show that the pathogen interferes with host pathways. The CISH protein triggers ubiquitination and degradation of H^{+}-V-ATPase, thus shutting down the proton pump.

Queval et al., 2017, Cell Reports *20*, 3188–3198
September 26, 2017 © 2017 The Authors.
http://dx.doi.org/10.1016/j.celrep.2017.08.101

CrossMark
CellPress

OPEN ACCESS
CellPress

Cell Reports
Article

Mycobacterium tuberculosis Controls Phagosomal Acidification by Targeting CISH-Mediated Signaling

Christophe J. Queval,[1,2] Ok-Ryul Song,[1,3] Jean-Philippe Carralot,[3] Jean-Michel Saliou,[1,4] Antonino Bongiovanni,[1] Gaspard Deloison,[1] Nathalie Deboosère,[1] Samuel Jouny,[1] Raffaella Iantomasi,[1] Vincent Delorme,[1,3] Anne-Sophie Debrie,[1] Sei-Jin Park,[3] Joana Costa Gouveia,[1] Stanislas Tomavo,[1,4] Roland Brosch,[2] Akihiko Yoshimura,[5] Edouard Yeramian,[6,*] and Priscille Brodin[1,3,7,*]

[1]University Lille, CNRS, Inserm, CHU Lille, Institut Pasteur de Lille, U1019–UMR 8204, CIIL–Center for Infection and Immunity of Lille, 59000 Lille, France
[2]Institut Pasteur, Unit for Integrated Mycobacterial Pathogenomics, 75015 Paris, France
[3]Institut Pasteur Korea, 16 Daewangpangyo-ro 712 beon-gil, Bundang-gu, Seongnam-si, Gyeonggi-do 463-400, South Korea
[4]Plateforme de Protéomique et Peptides Modifiés (P3M), CNRS, Institut Pasteur de Lille, University Lille, 59000 Lille, France
[5]Department of Microbiology and Immunology, Keio University School of Medicine, 35 Shinanomachi, Shinjyuku-ku, Tokyo 160-8582, Japan
[6]Unité de Microbiologie Structurale, CNRS UMR3528 Institut Pasteur, 75015 Paris, France
[7]Lead Contact
*Correspondence: edouard.yeramian@pasteur.fr (E.Y.), priscille.brodin@inserm.fr (P.B.)
http://dx.doi.org/10.1016/j.celrep.2017.08.101

SUMMARY

Pathogens have evolved a range of mechanisms to counteract host defenses, notably to survive harsh acidic conditions in phagosomes. In the case of *Mycobacterium tuberculosis*, it has been shown that regulation of phagosome acidification could be achieved by interfering with the retention of the V-ATPase complexes at the vacuole. Here, we present evidence that *M. tuberculosis* resorts to yet another strategy to control phagosomal acidification, interfering with host suppressor of cytokine signaling (SOCS) protein functions. More precisely, we show that infection of macrophages with *M. tuberculosis* leads to granulocyte-macrophage colony-stimulating factor (GM-CSF) secretion, inducing STAT5-mediated expression of cytokine-inducible SH2-containing protein (CISH), which selectively targets the V-ATPase catalytic subunit A for ubiquitination and degradation by the proteasome. Consistently, we show that inhibition of CISH expression leads to reduced replication of *M. tuberculosis* in macrophages. Our findings further broaden the molecular understanding of mechanisms deployed by bacteria to survive.

INTRODUCTION

Biological acids play key roles in the innate immune responses of eukaryotic hosts. To counteract acid-mediated defense mechanisms, encountered at extracellular or intracellular levels, pathogens have evolved a range of strategies. In the extracellular context, *Escherichia coli*, *Vibrio cholerae*, or *Salmonella typhimurum* have elaborated sophisticated strategies to survive in the harsh acidic conditions of gastrointestinal tracts (Foster, 2004). In the intracellular context, since Metchnikoff (who reported in 1905 that acidic reactions can be observed in the phagosomes of guinea pig macrophages upon ingestion of bacteria), numerous studies have elucidated mechanisms used by pathogens to overcome acid-mediated defense strategies of macrophages (Kaufmann, 2008). Pathogens use different strategies to avoid or to resist to progressive phagosomal maturation, either interfering with phagosomal trafficking or adapting to the harsh environment of the phagosome (Flannagan et al., 2009). For example, *Listeria monocytogenes* secretes pore-forming listeriolysin, which induces a rapid collapse of the phagosomal membrane, thereby promoting bacterial escape into the cytosol (Hamon et al., 2006). *Coxiella burnetii* modifies its gene expression to undergo active replication in large acidic vacuoles (McDonough et al., 2013). For *Mycobacterium tuberculosis* (*Mtb*), intracellular survival requires the regulation of pH homeostasis inside the vacuole, stabilizing the phagosomal acidification around pH 6.3–6.5. It was previously suggested that, in this case, such stabilization is caused by a defective retention of the V-ATPase complex at the phagosome (Pethe et al., 2004; Sturgill-Koszycki et al., 1994). Accounting for this phenomenon, it was shown that the microbial tyrosine phosphatase PtpA (Rv2234) prevents the tethering of V-ATPase to the *Mtb*-containing vacuole (Wong et al., 2011). More broadly, many mycobacterial products have been shown to arrest phagosome maturation and acidification, such as lipoarabinomannan, trehalose dimycolate, or acyltrehalose-containing glycolipids (Brodin et al., 2010; Pethe et al., 2004; Philips and Ernst, 2012). Whereas it is considered that the *Mtb* phagosome remains immature (Armstrong and Hart, 1971; Russell, 2001), studies showed that *Mtb* is able to induce phagosomal rupture at later stages of infection (Simeone et al., 2012; van der Wel et al., 2007). This capacity depends on a functional ESX-1 type VII secretion system and requires control of phagosomal acidification (Simeone et al., 2015). These findings shed new light on the multifaceted question of how *Mtb* counteracts phagosomal acidification. Altogether, these observations hint to the possible existence of alternative mechanisms, which could be used by pathogenic mycobacteria to block phagosomal acidification.

To address this important question, we investigate here the various macrophage pathways susceptible to being manipulated by *Mtb* during infection, resorting to genome-wide RNAi

 © 2017 The Authors.
This is an open access article under the CC BY license (http://creativecommons.org/licenses/by/4.0/).

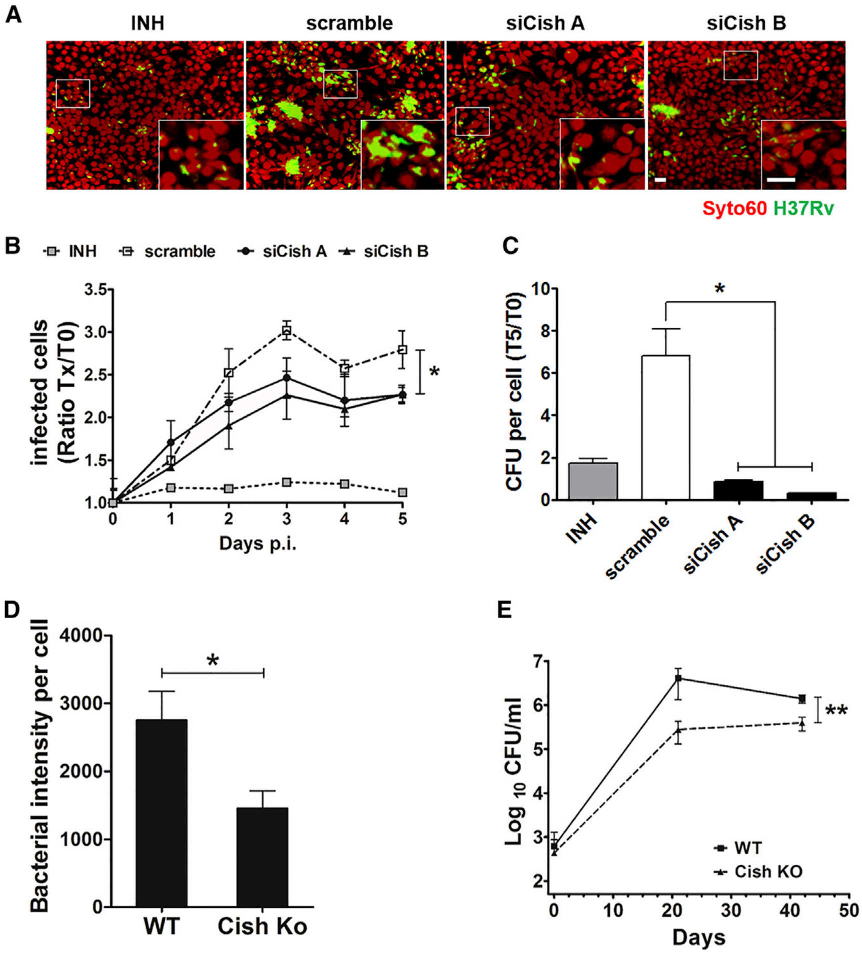

Figure 1. Cish Promotes *Mtb* Replication

(A) Typical confocal microscope images obtained in macrophages transfected with non-targeting siRNA controls (scramble) or Cish siRNA duplexes (siCishA or siCishB) or treated with 5 μg/mL of INH at 5 days p.i. with H37Rv-GFP. SYTO60-labeled cells are in red, and H37Rv-GFP bacteria are in green. Scale bar, 5 μm.

(B) Quantification of *Mtb* H37Rv intracellular growth in macrophages silenced for Cish with siRNA duplexes A (black solid circles on solid lines) and B (black solid triangles on solid lines). Positive controls (scramble siRNA + INH, gray solid squares on dotted lines) and negative controls (scramble siRNA, gray open squares on dashed lines), as assessed by monitoring the percentage of infected RAW 264.7 cells. For each time point, the percentage was normalized to that observed at 5 hr p.i. (T0 p.i.). Reported values represent fold changes (Tx/T0) ± SD.

(C) CFU numbers at 5 days p.i. normalized to the number of macrophages (T5). Results are means ± SD from representative experiments performed in duplicate. Positive control: INH, 5 μg/mL.

(D) Image-based quantification of the intracellular replication of H37Rv-GFP at 5 days p.i. in BMDM from Cish KO and WT mice.

(E) CFU determination in the lungs of Cish KO and WT mice challenged with *Mtb* H37Rv at 0, 21, and 42 days. Quantification represents the average of log of CFUs in lung ± SD. n = 4 mice per group per time point. Experiment was performed twice.

$^{*}p < 0.05$, $^{**}p < 0.01$.

high-content screening in *Mtb*-infected macrophages. By this approach, we identified cytokine-inducible SH2-containing protein (CISH) as a pivotal host factor contributing to the growth of *Mtb* in macrophages. CISH was originally identified as the first member of the suppressor of cytokine signaling (SOCS) family of proteins, which comprises eight members (CISH and SOCS1 to SOCS7) that are all key immunity regulators known to control cytokine signaling by inhibiting JAK/STAT activity (Yoshimura et al., 1995, 2007, 2012). We further show that entry of *Mtb* in macrophages induces rapid release of granulocyte-macrophage colony-stimulating factors (GM-CSFs), triggering STAT5 signaling and leading to early CISH expression. Moreover, we demonstrate an enrichment of CISH around *Mtb*-containing phagosomes, with the protein targeting the catalytic subunit A of V-ATPase (ATP6V1A) for ubiquitination, thus promoting its degradation.

RESULTS

CISH Promotes *Mtb* Replication

To study the effect of Cish on intracellular replication of *Mtb*, we used the visual phenotypic assay in murine macrophages (RAW 264.7) that relies on the automated monitoring, by confocal fluorescent microscopy, of intracellular growth of GFP-expressing *Mtb* H37Rv (H37Rv-GFP) (Christophe et al., 2009; Queval et al., 2014). Macrophages were first transfected with two small interfering RNAs (siRNAs) targeting Cish following established protocols (Carralot et al., 2009) and then infected with H37Rv-GFP. Cells were further incubated for 5 days before counterstaining with whole-cell dye SYTO60 and were analyzed with image acquisition using an Opera automated confocal microscope. Non-targeting siRNAs (scramble siRNAs) alone and in combination with isoniazid (INH) were used as negative and positive controls, respectively (Figure 1A). Customized image analysis was used for the quantification of relevant parameters, such as the number of macrophages, the percentage of infected cells, and the bacterial area per infected cell (Christophe et al., 2009; Queval et al., 2014). A decrease in the percentage of infected cells for Cish-silenced samples relative to non-targeting controls was observed, suggesting that Cish either inhibits *Mtb* intracellular replication or contributes to increased death of *Mtb* (Figures 1A and 1B). The role of Cish in promoting bacterial replication was confirmed by the relative numbers of colony-forming units (CFUs) per cell, with a decrease in the

OPEN ACCESS
CellPress

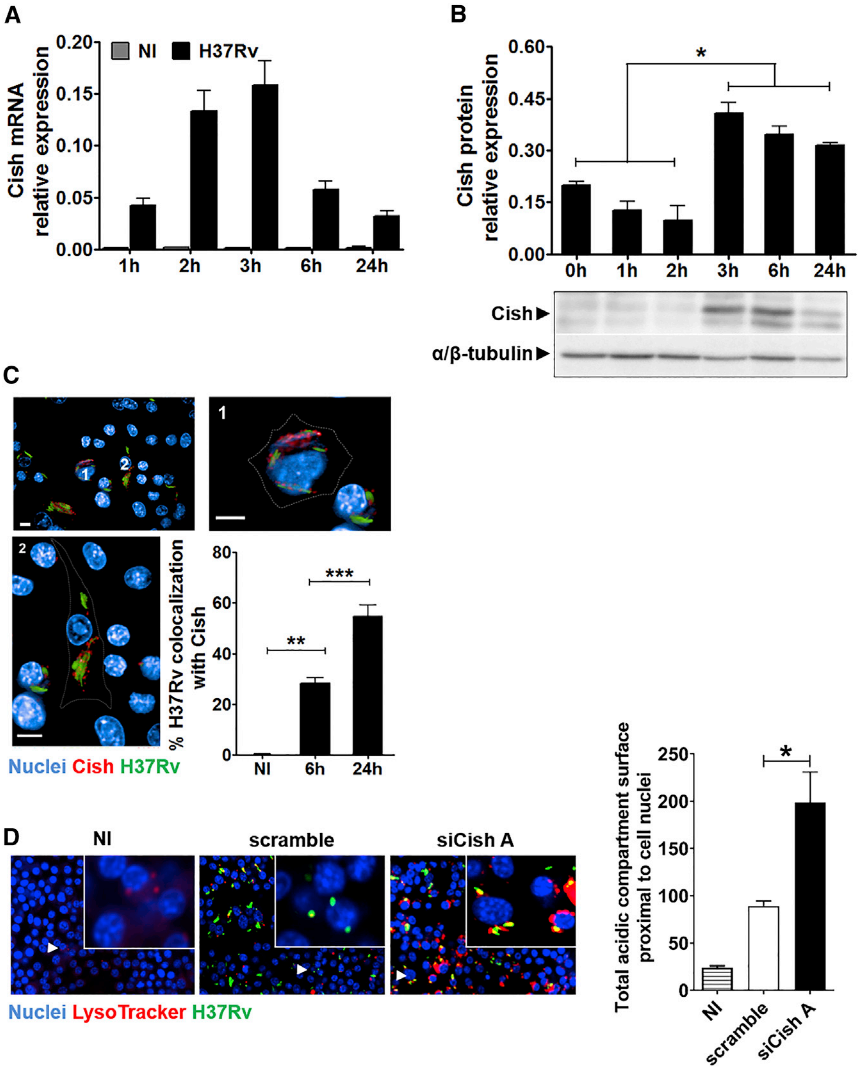

Figure 2. Cish Is Recruited at the *Mtb* Vacuole and Interferes with Phagosome Acidification

(A) Cish mRNA levels in RAW 264.7 macrophages infected with *Mtb* H37Rv (black bars) compared with non-infected (NI) ones (gray bars). Results are means ± SD from representative experiments performed in triplicate.

(B) Cish expression analysis by immunoblotting. α/β-tubulin served as internal controls.

(C) Analysis of the intracellular localization of Cish by fluorescence microscopy. Nuclei were labeled with DAPI (blue), and Cish was immunolabeled using Cish antibody (red). *Mtb* H37Rv-GFP is in green. Plot represents the percentage of *Mtb* H37Rv-GFP bacteria that colocalize with Cish.

(D) Typical images and related quantification of phagosome acidification of NI, *Mtb* H37Rv-GFP-infected macrophages transfected with scramble control siRNA (scramble), or Cish siRNA duplexes (siCishA). DAPI-labeled cell nuclei are in blue, *Mtb* H37Rv-GFP is in green, and acidic-pH-sensitive LysoTracker staining is in red. The LysoTracker signal was set to minimum in NI controls.

Scale bars, 10 μm. *p < 0.05, **p < 0.01, ***p < 0.001.

numbers of viable bacteria per cell for Cish-silenced samples relative to scramble controls (Figure 1C). Corroborating these results, the bacterial load at 5 days post-infection (p.i.) was about 2-fold lower in bone marrow-derived macrophages (BMDMs) from Cish knockout (KO) mice than in BMDMs from wild-type (WT) mice (Figure 1D).

We found that in an in vivo murine infection model, the number of *Mtb* CFUs in the lungs of Cish KO mice was lower than that in WT control animals (Figure 1E). Although soon after intranasal challenge with *Mtb* H37Rv, the colonization of the lungs was found to be similar for infected Cish WT mice, the numbers of CFUs in lungs were 10-fold lower for Cish KO mice relative to WT mice during the acute replication phase. These results point to a counter-intuitive role for this host protein in promoting *Mtb* replication in the early stage of infection. We next decided to assess whether CISH is also required for *Mtb* survival in human macrophages. We first ascertained that our reverse transfection of pooled siRNAs targeting CISH did not induce cytotoxicity and validated the silencing efficiency by qRT-PCR (Figures S1A and S1B). The intracellular bacterial load upon *Mtb* infection was then quantified as earlier (Christophe et al., 2009; Queval et al., 2014, 2016). The bacterial load at 3 hr p.i. was the same for small interfering CISH (siCISH) human macrophages and for scramble control, whereas at 4 days p.i., the bacterial load in siCISH human macrophages was two times lower than that in controls (Figure S1C). These results strongly suggest that CISH plays a role in enhancing the intracellular replication of *Mtb*, both in murine and human macrophages and in an in vivo mouse infection model.

CISH Is Recruited at the *Mtb* Vacuole and Interferes with Phagosome Acidification

To understand how CISH promotes *Mtb* replication, we first quantified the expression of Cish. Cish was absent in naive macrophages, while its transcription was rapidly upregulated upon infection, with a peak of expression at 3 hr p.i. (Figure 2A). This result is consistent with a previous report of increased Cish expression in murine primary macrophages infected with *Mtb* (Koo et al., 2012). Accordingly, expression of Cish was detected 3 hr p.i. by western blot and maintained up to 24 hr (Figure 2B). Immunofluorescence studies revealed that Cish was recruited at the *Mtb*-containing vacuole with about 30% of phagosomal bacteria positive for Cish 6 hr p.i. and 55% after 24 hr (Figure 2C). A similar accumulation of CISH on phagosomes was seen in human macrophages. CISH was also recruited at the phagosome

by dead *Mtb*; however, only live *Mtb*-infected human macrophages retained CISH at the vacuole at 24 hr p.i. (Figures S2A and S2B). The question then arises whether CISH can play a role in the inhibition of phagosome maturation. We thus monitored the fusion of *Mtb* phagosomes with lysosomes by fluorescence microscopy, using the pH-sensitive LysoTracker dye. It was demonstrated previously that the intensity of LysoTracker-labeling directly correlates with the presence of acidic lysosomal milieu in *Mtb* vacuoles (Brodin et al., 2010). Upon *Mtb* infection, the LysoTracker intensity was significantly higher in macrophages silenced for Cish than that in scramble (Figure 2D), strongly suggesting that Cish interferes with the process of phagolysosomal fusion. The existence of a link between accumulation of CISH and restriction of phagosomal acidification was confirmed in siCISH *Mtb*-infected human macrophages upon monitoring mean pHrodo intensity levels per *Mtb*-containing phagosomes (Figure S2C). Compared to scramble human macrophages, bacterial phagosomes from siCISH human macrophages exhibited a significant increase in pHrodo fluorescence intensity, indicative of stronger phagosomal acidification in CISH-silenced cells (Figures S2D–S2G). Most mycobacterial phagosomes in scramble human macrophages displayed a characteristic pH between 6 and 7, whereas in siCISH human macrophages, most of the phagosomes appeared more acidic, with a pH between 5 and 6 (Figure S2H).

We then wanted to pinpoint the mechanism underlying such an effect of CISH. Because CISH has the ability to ubiquitinate proteins, targeting them for the ubiquitin-dependent proteasomal degradation (Piessevaux et al., 2008; Yoshimura et al., 2007), we investigated the effect of CISH on protein ubiquitination and proteasome activity. We assessed such an impact at 24 hr p.i. when CISH accumulated mostly around *Mtb*-containing phagosomes. siCISH *Mtb*-infected human macrophages were then labeled for poly-ubiquitin motifs, and the mean levels of poly-ubiquitin signals per cell were assessed using dedicated image-analysis software. Such analyses revealed that siCISH human macrophages exhibited a strong decrease in poly-ubiquitin fluorescence intensity compared to scramble ones (Figures 3A and 3B), showing that CISH induced ubiquitination of proteins. Because protein poly-ubiquitination leads to proteasomal degradation, we questioned whether the decrease of poly-ubiquitin signals in siCISH macrophages was associated with a decreased eukaryotic proteasome activity. The proteasome activity in *Mtb*-infected human macrophages was thus monitored by quantification of the cleavage of fluorogenic LLVY-R110 proteasome substrates (Figure S3). We found that the proteasome activity was significantly decreased in siCISH conditions, relative to scramble (Figure 3C). Given the involvement of CISH in ubiquitination and proteasome activities in *Mtb*-infected macrophages, we therefore wanted to identify the proteins susceptible to be targeted by CISH for proteasomal degradation.

CISH Targets ATP6V1A for Ubiquitination

We implemented a high-throughput proteomic approach to identify proteins ubiquitinated by CISH, comparing the set of ubiquitinated proteins found in *Mtb*-infected siCISH human macrophages with that found in scramble ones. For each sample, ubiquitinated proteins were first purified by pull-down of total protein extracts with the UbiQapture-Q matrix and then analyzed using tandem mass spectrometry (Figure S4A). Based on this analysis, 1,282 ubiquitinated proteins were detected in siCISH samples, compared to 1,556 proteins in scramble samples. A more stringent comparison, with scoring taking into account the number of peptides and spectra in the tandem mass spectrometry, allowed the selection of 65 potential CISH targets, with the corresponding proteins being significantly less abundant in the CISH-silenced conditions relative to scramble controls (Figures S4B and S4C; Table S1). Among the CISH-targeted proteins, we identified ATP6V1A, which is part of the V-ATPase multi-subunit complex that had previously been shown not to be present at the non-acidified *Mtb* phagosome (Sturgill-Koszycki et al., 1994; Sun-Wada et al., 2009). We thus tested whether the ubiquitination of ATP6V1A by CISH could interfere with the acidification process. First, we confirmed by immunoprecipitation the ubiquitination of ATP6V1A, as seen 24 hr after *Mtb* infection (Figure 3D). To avoid proteasomal degradation that could impair the detection of ATP6V1A, macrophages were treated for 5 hr with proteasome inhibitor MG132 before cell lysis. In such conditions, ubiquitin signals were not detected in siCISH samples, in contrast to scramble controls (Figure 3D).

We next compared the distributions of CISH and ATP6V1A specifically at the level of *Mtb*-containing phagosomes. Confocal image acquisition of infected macrophages revealed a patchy distribution for ATP6V1A throughout cells (Movie S1). At the phagosome, CISH and ATP6V1A display largely superimposable fluorescence intensity profiles (Figure 3E). A quantitative analysis on a large set of phagosomes led to a significant correlation between the distribution of CISH and that of ATP6V1A (Figure 3F). This correlation takes into account the distribution of the two proteins around the phagosomes. In addition, the correlation coefficient increased to a value larger than 0.4 upon treatment with proteasome inhibitor MG132, suggesting that ubiquitination of ATP6V1A by CISH leads to its degradation by the proteasome.

CISH activity was previously shown to be mediated by its C-terminal SOCS box domain, which is essential for the interaction of CISH with various proteins, as well as with the Cullin-RING E3 ubiquitin ligase complex, leading to the ubiquitin labeling of target proteins for proteasomal degradation (Kamura et al., 2004; Masuhara et al., 1997; Piessevaux et al., 2008; Zhang et al., 1999). For example, CISH has been shown to induce the degradation of growth hormone receptor, erythropoietin receptor, and Bcl-2-interacting mediator of cell death extra long (BimEL) (Landsman and Waxman, 2005; Verdier et al., 1998; Zhang et al., 2008). In this general structural background, we wanted to ascertain (1) the direct physical interaction of CISH with intracellular ATP6V1A and (2) the proteasomal degradation of ATP6V1A. First, to assess the physical interactions between CISH and ATP6V1A, we resorted to HEK293 cells that we engineered to overexpress murine Cish or CishΔSOCS box (Figure 4A). To get a comparable amount of proteins in the two samples, cells were first treated with proteasome inhibitor MG132 for 5 hr before lysis. Endogenous ATP6V1A was then immunoprecipitated and Cish was detected by western blot (Figure 4B; Figures S5A and S5B). Cish was found complexed to ATP6V1A in samples overexpressing Cish, but not in control ones. In contrast, we found that in the absence of the SOCS box domain,

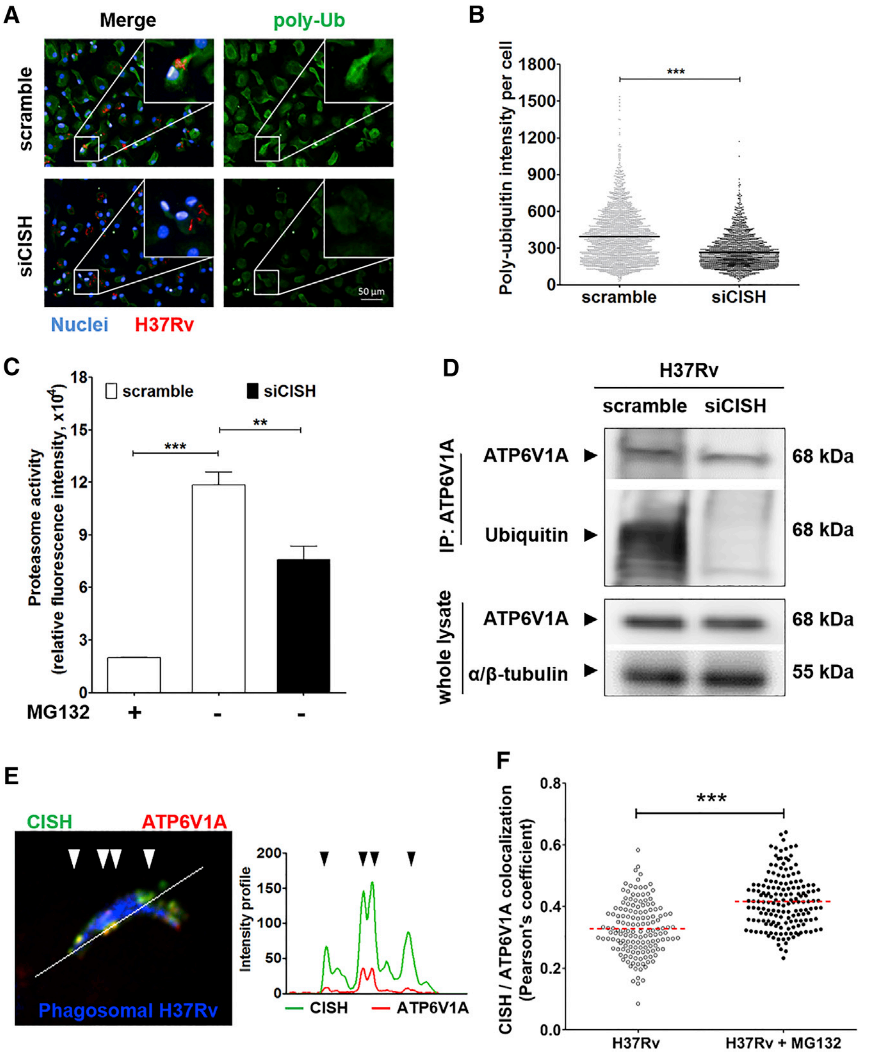

Figure 3. CISH Targets the Subunit A of V-ATPase for Ubiquitination

(A) Typical confocal microscope images of *Mtb*-infected human macrophages that were labeled with antibody targeting poly-ubiquitin (poly-Ub) motifs. DAPI-labeled cell nuclei are in blue, *Mtb* H37Rv-DsRed is in red, and poly-ubiquitin conjugated proteins are in green. White squares delimitate the area magnified in the upper-right corners of the various micrographs. Scale bar, 50 μm.

(B) Mean poly-ubiquitin signal intensity per cell. Plotted values are for siCISH sample cells (N = 1,900 cells) and scramble controls (N = 2,400 cells).

(C) Monitoring proteasome activity in siCISH and scramble samples upon infection with *Mtb* H37Rv by quantification of fluorescence, resulting from the cleavage of LLVY-R110. As negative controls, scramble human macrophages were treated for 4 hr with 1 μM MG132 proteasome inhibitor (MG132 +).

(D) Ubiquitination profile of ATP6V1A in siCISH and scramble human macrophages 24 p.i. by *Mtb* H37Rv monitored by western blot. 5 hr before lysis, cells were incubated in the presence of 1 μM MG132 and ATP6V1A was immunoprecipitated from whole-cell lysate (top panel). As control, the amount of ATP6V1A in whole-cell lysate was ascertained by probing with anti-ATP6V1A and anti-α/β-tubulin (lower panel).

(E) Intracellular localization of CISH and ATP6V1A analyzed by confocal microscopy in human macrophages. H37Rv-GFP bacteria are in blue, CISH is in green, and ATP6V1A is in red. The white line represents a section of the picture used to quantify the intensity of fluorescence for both CISH and ATP6V1A. Values of intensities of CISH (green) and ATP6V1A (red) are plotted next to the micrograph.

(F) Intracellular colocalization of CISH and ATP6V1A, analyzed by fluorescence confocal microscopy in *Mtb*-infected human macrophages treated with DMSO (H37Rv) or 1 μM MG132 for 5 hr. CISH and ATP6V1A colocalization was assessed by calculating Pearson's correlation between CISH and ATP6V1A fluorescence intensities around phagosomes (N = 150 phagosomes).

All micrographs and plots are representative of two independent experiments, each made in duplicate. $^{**}p < 0.01$, $^{***}p < 0.001$.

the binding of Cish to ATP6V1A was strongly weakened, suggesting that the SOCS box domain is required for an optimal binding (Figure 4B). Second, to ascertain the link between Cish and ATP6V1A degradation, we compared the respective intracellular amounts of ATP6V1A in HEK-expressing Cish or CishΔ SOCS box in the absence of the proteasome inhibitor (Figure 4C). These comparisons showed that the amount of intracellular ATP6V1A was drastically lower in HEK-expressing Cish than in HEK control (pcDNA), confirming that Cish modulates the degradation of ATP6V1A. Moreover, in the absence of the Cish SOCS box domain, ATP6V1A was detectable in large amounts in cell lysates, demonstrating that the expression of functional Cish is required for the degradation of ATP6V1A (Figure 4C). Finally, we performed experiments to assess whether the degradation of ATP6V1A was mediated by the proteasome. To this end, HEK-expressing Cish samples were treated with proteasome inhibitor MG132 at different time points. In the presence of MG132, both Cish and ATP6V1A accumulated within cells, suggesting that the intracellular turnover of these two proteins was mediated by the proteasome (Figure 4D). From these results, a mechanistic model can be drawn for the inhibition of phagosomal acidification, in which CISH interferes with V-ATPase through the ubiquitination and subsequent proteasomal degradation of its subunit A.

Expression of CISH in *Mtb*-Infected Macrophages Depends on STAT5 Activation

We then investigated the regulation of CISH expression to further elucidate the CISH-associated signaling cascade targeted by *Mtb*. It was reported that the regulation of cell functions involving SOCS proteins is closely connected with the activities of proteins of the STAT family (Yoshimura et al., 1995, 2007). In particular, STAT5 and CISH belong to the same negative regulatory loop, because STAT5 can bind the *CISH* promoter region, thereby

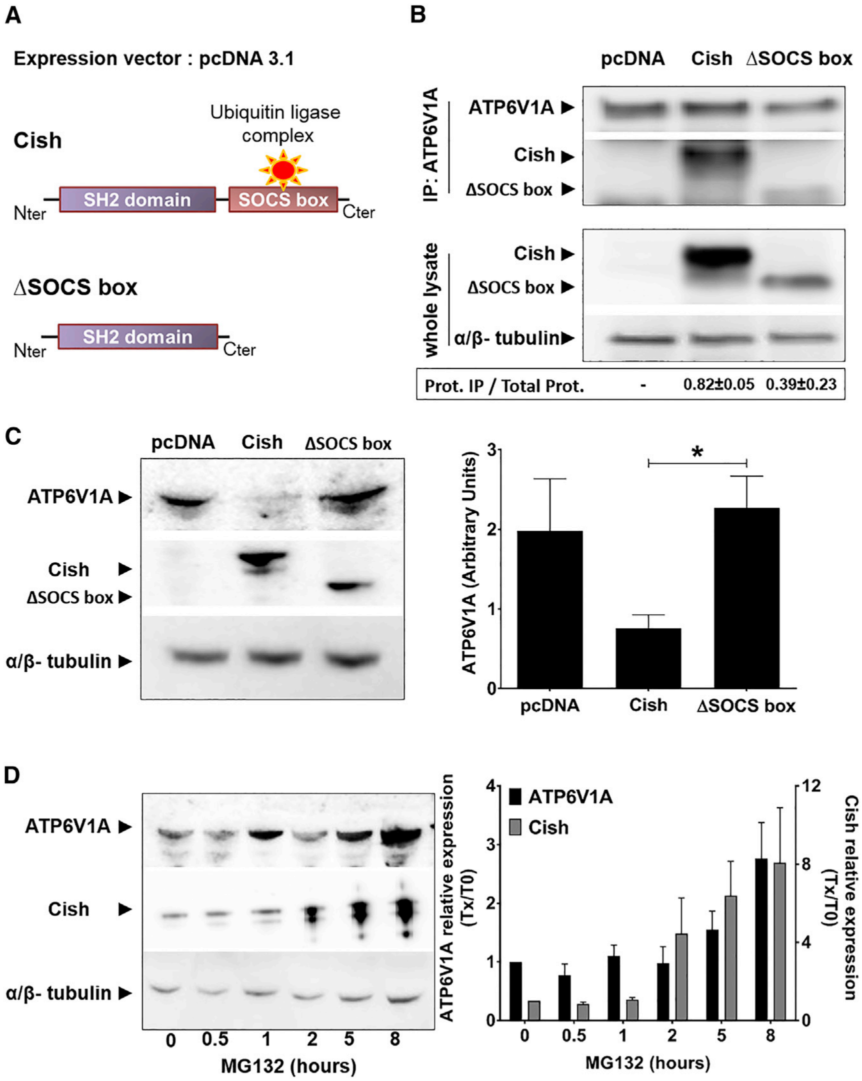

Figure 4. Ubiquitination of ATP6V1A by Cish Leads to Its Subsequent Proteasomal Degradation

(A) Schematic representations of Cish full sequence (Cish) and Cish deleted from its SOCS box domain (ΔSOCS box).

(B) Pull-down of Cish following immunoprecipitation of ATP6V1A from HEK293 cells that overexpress Cish or CishΔSOCS box. An empty vector is used as the negative control (pcDNA). In the lower panel, Cish and CishΔSOCS expression from whole-cell lysates were ascertained by probing with anti-Cish and anti-α/β-tubulin (lower panel).

(C) Immunodetection of ATP6V1A from pcDNA, Cish, and CishΔSOCS box-overexpressing HEK cells. ATP6V1A was detected by probing with anti-ATP6V1A, and Cish and CishΔSOCS box were detected by probing with anti-Cish. Probing with anti-α/β-tubulin antibodies was used to confirm gel loading. Reported values represent the means of relative expression of ATP6V1A $\pm$ SEM from three independent experiments. *$p < 0.05$.

(D) Time-dependent accumulation of Cish and ATP6V1A in the presence of 5 μM MG132 proteasome inhibitor. For the quantification (right panel), both ATP6V1A expression and Cish expression were normalized to T0. Reported values represent fold changes (Tx/T0) $\pm$ SEM.

inducing CISH expression (Rascle and Lees, 2003). Accordingly, we first characterized the early activation of STAT5 during infection of human macrophages with *Mtb*. The amount of phosphorylated STAT5 proteins (PY-STAT5) was quantified by western blot up to 48 hr p.i., and the results showed a strong phosphorylation of STAT5 at 3 hr p.i. (Figure 5A; Figure S6A). Corroborating this result, the nuclear translocation of PY-STAT5 proteins in *Mtb*-infected human macrophages was monitored by indirect immunofluorescence and image-based quantification, showing that about 26% of human macrophages displayed nuclear translocation of PY-STAT5 proteins 3 hr p.i. (Figure 5B). We also found that PY-STAT5 proteins accumulate within nuclei of non-infected bystander cells, suggesting that STAT5/CISH signaling is effective in the entire macrophage population in our in vitro cell-based assay model.

We next investigated the impact of STAT5 downregulation on the production of CISH mRNA by qRT-PCR (Figure S6B). Five hours after infection with *Mtb*, human macrophages silenced for STAT5 (siSTAT5) failed to induce CISH mRNA production (Figure 5C), thus indicating that STAT5 acts as a transcriptional regulator of *CISH* in macrophages.

STAT5-Mediated CISH Expression Depends on Early Release of GM-CSF by *Mtb*-Infected Macrophages

Finally, we wanted to identify the signal triggering the activation of STAT5 by *Mtb* in human macrophages. Because STAT proteins are known to be activated through cytokine signaling, we assessed whether such activation mechanisms occurred in our settings. STAT5 activation was thus investigated in naive human macrophages stimulated with supernatants recovered from *Mtb*-infected human macrophages. This approach revealed that STAT5 was activated in supernatant-stimulated human macrophages, which displayed phosphorylation levels similar to those observed in *Mtb*-infected macrophages (Figure S7A). We also confirmed the nuclear translocation of STAT5 in naive human macrophages that were stimulated with supernatants recovered from *Mtb*-infected human macrophages (Figure S7B). In contrast, no nuclear translocation of STAT5 was detectable in naive human macrophages incubated with supernatants from non-infected cells. Overall, approximately 40% of human macrophages displayed nuclear translocation of STAT5 proteins after 1.5 hr of stimulation with supernatants from infected cells. This result suggests the occurrence of a rapid *Mtb*-dependent activation of STAT5 signaling that is mediated through stimulation of molecules released by infected macrophages. In this respect, GM-CSF was previously

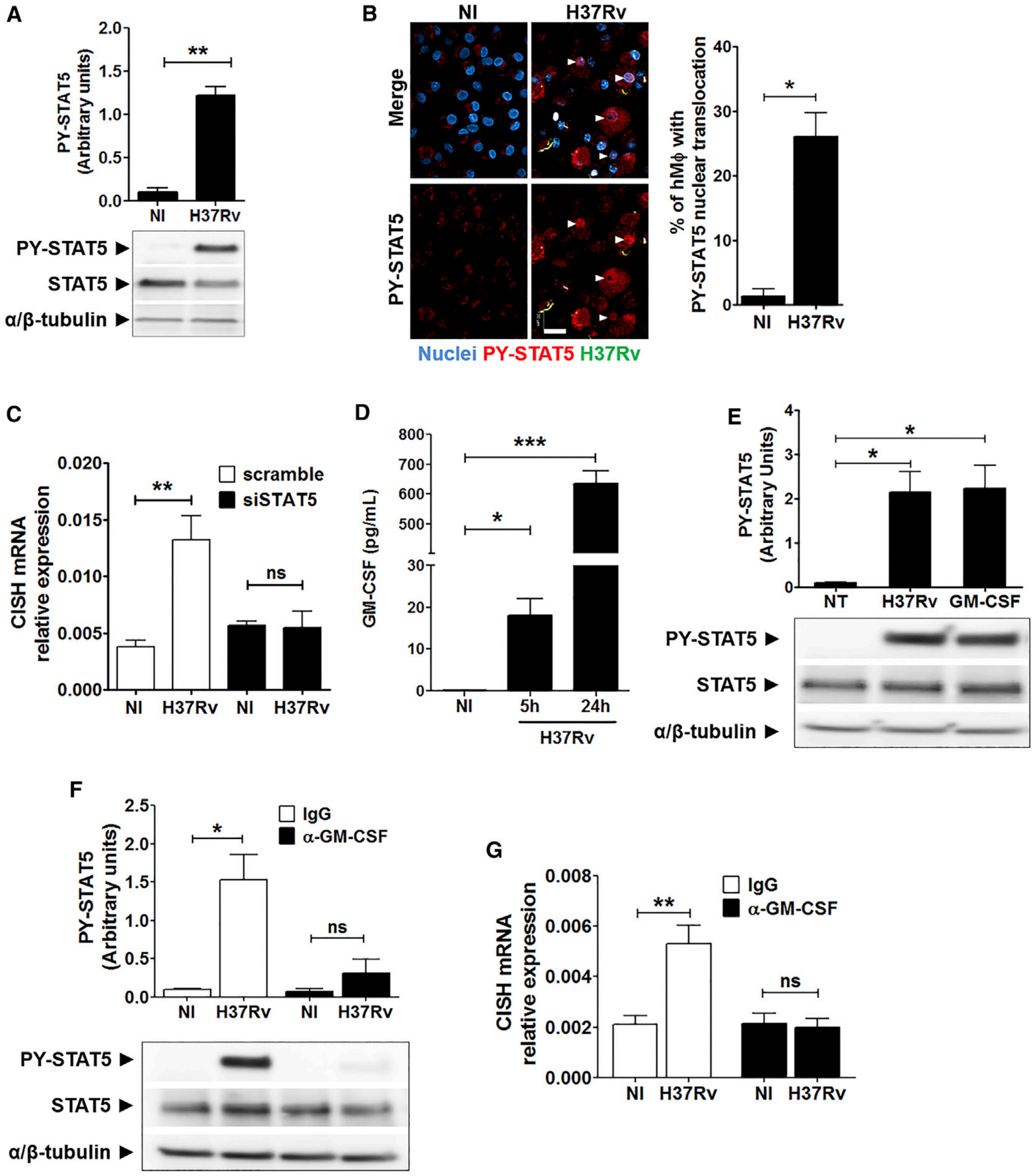

Figure 5. Expression of CISH in *Mtb*-Infected Macrophages Depends on GM-CSF-Mediated STAT5 Activation

(A) Analysis of STAT5 activation by immunoblotting of *Mtb*-infected human macrophages using anti-PY^{694}-STAT5. Probing with anti-STAT5 and anti-α/β-tubulin antibodies was used to confirm gel loading. Immunoblots are representative of three independent experiments with three donors.

(B) Typical images and quantifications of the nuclear translocation of PY-STAT5 analyzed by indirect immunofluorescence. The white arrows labeled the nuclei positive for PY-STAT5 staining. Scale bar, 20 μm. DAPI-labeled nuclei are in blue, H37Rv-GFP is in green, and PY-STAT5 is in red. Reported values represent the mean ± SD of the percentage of cells displaying STAT5 nuclear translocation. Data are representative of two independent experiments with two donors.

(C) CISH mRNA expression in *Mtb* H37Rv-GFP-infected siSTAT5 and scramble human macrophages (H37Rv) for 4 hr. Non-infected (NI) cells were used as control. The data shown are the means ± SD of relative CISH mRNA expression from three independent experiments.

(D) Quantification of GM-CSF release from supernatants collected from *Mtb* H37Rv-GFP-infected human macrophages for 5 or 24 hr. Reported values represent the average concentrations of cytokine released ± SEM from two donors, each tested in duplicate.

(E) STAT5 activation was analyzed by immunoblotting using anti-PY^{694}-STAT5 antibody. As positive control, human macrophages were treated with 50 ng/mL of human GM-CSF for 3 hr. Probing with anti-STAT5 and anti-α/β-tubulin antibodies was used to confirm gel loading.

(F) Effect of neutralizing anti-GM-CSF (α-GM-CSF) on STAT5 activation in *Mtb*-infected human macrophage by western blot analysis. IgG was used as control (10 μg/mL). Reported values represent the relative STAT5 phosphorylation ± SEM. Immunoblots are representative of three independent experiments performed with human macrophages from three donors.

(G) Effect of neutralizing anti-GM-CSF on CISH mRNA expression. The data correspond to the means ± SD of relative CISH mRNA expression from three independent experiments.

$*p < 0.05$, $**p < 0.01$, $***p < 0.001$. NS, not significant.

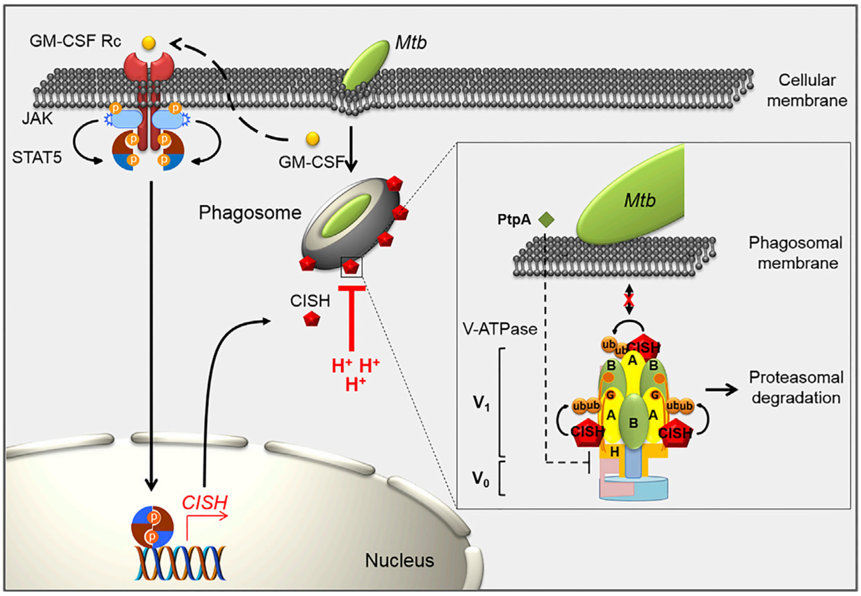

Figure 6. Working Model for Explaining CISH-Mediated Limitation of Acidification of Mycobacterial Phagosomes through Targeting ATP6V1A for Ubiquitination and Proteasomal Degradation

Proposed mechanism by which *Mtb* co-opts STAT5-mediated expression of CISH to promote V-ATPase degradation in macrophages. Right after *Mtb* uptake, early release of GM-CSF induces autocrine activation of STAT5 via the GM-CSF receptor (GM-CSF Rc). Next, activated STAT5 translocates into nucleus to induce CISH expression. CISH is then recruited and retained at the mycobacterial phagosome, where it represses phagosomal acidification by targeting ATP6V1A for ubiquitination. V-ATPases are composed of an ATP-hydrolytic domain (V_1) and a proton-translocation domain (V_0), acting together as a rotary machine. CISH binds and mediates ubiquitination of ATP6V1A, enabling its proteasomal degradation. The cytosolic V-ATPase was already shown to be the target of the mycobacterial tyrosine phosphatase PtpA (Wong et al., 2011) via subunit H, suggesting that CISH and PtpA may act in synergy for an efficient blockade of phagosomal acidification.

shown to act as a possible inducer of STAT5 signaling in macrophages (Lehtonen et al., 2002). Accordingly, we first checked whether GM-CSF was secreted by human macrophages infected with *Mtb* in our settings and GM-CSF accumulates during the first 24 hr upon infection (Figure 5D). Next, we showed that addition of purified GM-CSF led to STAT5 phosphorylation in naive non-infected human macrophages (Figure 5E). The observed effect was blocked by the use of anti-GM-CSF (α-GM-CSF) antibodies. Such antibodies strongly impaired STAT5 activation upon infection, while PY-STAT5 levels remained unaffected in infected cells treated with control immunoglobulin G (IgG) (Figure 5F). These results strongly suggest that early secretion of GM-CSF, induced by the uptake of *Mtb* into macrophages, leads to a specific activation of STAT5 signaling. In addition, we found that treatment of cells with GM-CSF triggered CISH mRNA expression (Figure S7C), whereas neutralization of GM-CSF during *Mtb* infection resulted in the inhibition of CISH mRNA production (Figure 5G). Altogether, these results thus allow us to propose a new complementary model of regulation for the prevention of phagosomal acidification by *Mtb* in macrophages (Figure 6). According to this new model, *Mtb* infection induces GM-CSF secretion, triggering a regulatory cascade with the nuclear translocation of STAT5 leading to the expression of CISH, which in turn ubiquitinates ATP6V1A in the immediate surrounding of *Mtb*-containing phagosomes, thus allowing the control of phagosomal acidification.

DISCUSSION

Since Metchnikoff, the elucidation of the mechanisms allowing pathogens to escape the acidic defense of macrophages represents an ongoing endeavor. During phagosome maturation, lysosomal V-ATPase, considered the key enzyme involved in the process, is rapidly recruited to the phagosomal membrane for luminal acidification (Sun-Wada et al., 2009). Because the acidification process represents a potent antimicrobial host defense, pathogenic invaders deploy an array of strategies to interfere with V-ATPase activity. For example, *Legionella pneumophila* controls phagosomal acidification through interactions of SidK with ATP6V1A (Zhao et al., 2017).

Here, we have identified a hitherto-unknown strategy developed by *Mtb* to target the V-ATPase complex, interfering with the signaling cascade of the SOCS family member CISH. As a result of this successful strategy, the phagosomal pH of *Mtb*-infected macrophages remains above the levels usually required for optimal activity of lysosomal digestive enzymes and production of reactive oxygen species (Sun-Wada et al., 2009; Vieira et al., 2002). However, previous work showed that such a lack of phagosomal acidification is also attributable to a defective fusion of the V-ATPase complex with the phagosomal membrane (Sturgill-Koszycki et al., 1994). This effect was later ascribed to the action of PtpA, reported to be secreted by *Mtb* through the phagosomal membrane and into the cytoplasm of the host macrophage (Wong et al., 2011). In this mechanism, PtpA binds to the subunit H of the cytosolic V-ATPase, thereby blocking its association with the phagosomal membrane and impeding phagosomal acidification. Several other mycobacterial factors have been also shown to affect early phagosome acidification in macrophages (Brodin et al., 2010). However, the molecular mechanisms underlying their action on host phagosomal proteins have not yet been precisely characterized, and it cannot be excluded that they may be involved in V-ATPase sorting.

It was considered until recently that in in vitro culture conditions, the *Mtb* phagosome remains immature for several days. Recent results obtained with sensitive flow cytometry-based cytosolic pattern recognition assays clearly suggest that *Mtb*-containing phagosomes develop membrane disruptions a few hours p.i. (Augenstreich et al., 2017; Simeone et al., 2015).

Such ESX-1 type VII secretion-dependent phagosomal rupture is consistent with the associated rapid triggering of type I interferon responses (Stanley et al., 2007) and requires inhibition of early phagosomal acidification (Majlessi and Brosch, 2015; Simeone et al., 2015).

In light of our results, it is possible that the blockade of phagosomal acidification, by shutting down the V-ATPase proton pump, may be potentiated by a synergic action between CISH and PtpA. PtpA has been described to bind cellular ubiquitin or the RING domain of the TRIM27 ubiquitin ligase (Wang et al., 2016). Thus, our findings open new questions and perspectives for research aiming to understand the possible mutual interactions between the two as-yet unconnected cellular events.

Our mechanistic model is schematically summarized in Figure 6, highlighting the host signaling pathway co-opted by the pathogen to shut down and counteract the acidification of the phagosome. The finding that the CISH protein is targeted by *Mtb* reveals a hitherto-unsuspected role for CISH, thereby providing handles for exploring mechanistic details of this previously uncharacterized antimicrobial activity of macrophages.

Our study mainly focused on the role of CISH in infected cells. In the absence of intracellular bacteria, CISH might inhibit cytokine responses in macrophages by shutting down the STAT5 signaling pathway, as is the case in T lymphocytes (Yoshimura et al., 1995). However, in infected macrophages, as shown here, CISH sequestration around bacteria-containing phagosomes appears to promote its activity at the phagosomal membrane. Thus, depending on the presence of intracellular bacteria, CISH-mediated processes might contribute to heterogeneous responses within the host cell population, a phenomenon that could be further investigated with single-cell approaches.

In a broader context, we could speculate that CISH-mediated control of acidification may be also used by other pathogens that have already been shown to exert effects on V-ATPase (Zhao et al., 2017). In this respect, increased susceptibility in individuals to various infectious diseases was linked to genetic polymorphisms in CISH (Khor et al., 2010). Finally, the coexistence of different (direct and indirect) mechanisms to counteract the antimicrobial action of macrophages is observed for other infectious agents, such as in infections by *Leishmania* spp. (Bhardwaj et al., 2010). It is intriguing that *Leishmania donovani* appears to have evolved an escape mechanism that interferes with the JAK-STAT signaling cascade of the host, although in this case, the interference concerns oxidative rather than acidic bursts and the pathway is used to inhibit the expression of inducible nitric oxide synthase (iNOS). Thus, the JAK-STAT signaling cascade represents a multifaceted host pathway that can be hijacked by different pathogens from different angles in the complex host-pathogen interaction relationships.

EXPERIMENTAL PROCEDURES

In Vitro Intracellular Assays

A recombinant strain of *Mtb* GFP-expressing *Mtb* H37Rv (H37Rv-GFP) was cultured as described in detail in the Supplemental Information. RAW 264.7 cells or primary human macrophages were reverse transfected with 50 nM of siRNA for 3 days before being infected with H37Rv-GFP as detailed in the Supplemental Information. Infection was allowed to proceed for 5 hr at 37°C and 5% CO_2, and extracellular bacilli were removed via extensive washing of the plate. Antibiotics were never used at any of the multiple steps of the experiments. Infected RAW 264.7 cells were incubated at 37°C and 5% CO_2 for 5 days. On day 5 after infection, cells were stained with 10 μM SYTO60 (Invitrogen) and confocal images were acquired using the automated fluorescence microscope Opera (PerkinElmer) as described previously (Christophe et al., 2009). Image analysis is described in detail in the Supplemental Information. For the quantification of phagolysosomal fusion, living cells were labeled with 1 μM LysoTracker red DND-99 (Invitrogen) and 10 μg/mL Hoechst 33342 (Sigma-Aldrich) for 1 hr at 37°C and 5% CO_2. For the quantification of phagosomal acidification by pHrodo, H37Rv-GFP was labeled with 0.5 mM amine-reactive pHrodo Red succinimidyl ester (Life Technologies) during 1 hr at 37°C in 100 mM sodium bicarbonate (pH 8.5) before cell infection. More details on these experiments are in the Supplemental Information.

Electrophoresis and Immunoblotting

After washing, cells were lysed and proteins were resolved by SDS-PAGE gels (Bio-Rad) and transferred onto a polyvinylidene difluoride (PVDF) membrane, which was probed with the appropriate antibodies as described in detail in the Supplemental Information.

Immunofluorescence for Image Based-Quantification of CISH, Polyubiquitin, ATP6V1A, and PY-STAT5

After infection, human macrophages were fixed with 10% neutral buffered formalin solution (HT5014, Sigma-Aldrich) for 30 min, permeabilized, and probed with the appropriate antibodies as described in detail in the Supplemental Information. For the quantification of CISH/V-ATPase colocalization around *Mtb* vacuole, confocal images were acquired using the confocal microscope Zeiss LSM880 and images were analyzed using ImageJ and the plugin JACoP (Bolte and Cordelières, 2006).

Quantification of Proteasome Activity

Proteasome activity was quantified using the Amplite Fluorimetric Proteasome 20S Activity Assay Kit from AAT Bioquest. Cells were incubated 3 hr with proteasome LLVY-R110 substrate as described in detail in the Supplemental Information (where appropriate, cells were treated for 4 hr with 1 μM MG132 proteasome inhibitor).

Pull-Down of Ubiquitinated Proteins and Detection of V-ATPase Catalytic Subunit A

Human macrophages were first transfected with siCISH or scramble before infection with H37Rv-GFP at a MOI of 1. Cells were lysed 24 hr p.i. Ubiquitinated proteins were then pulled down using the UbiQapture kit from Enzo Life Sciences, and proteomic analysis was performed as described in detail in the Supplemental Information. For detection of endogenous V-ATPase catalytic subunit A (ATP6V1A), lysates of H37Rv-GFP-infected human macrophages were incubated for 5 hr at 4°C with 30 μL of Protein A Sepharose CL-4B beads (GE Healthcare) in the presence of rabbit anti-ATP6V1A antibody (Proteintech Europe). After electrophoresis by SDS-PAGE and transfer into the PVDF membrane, ATP6V1A was immunoblotted using rabbit anti-ATP6V1A (Novus) and secondary antibody easy blot anti-rabbit IgG conjugated with horseradish peroxidase (HRP) (GeneTex). Ubiquitin was revealed using ubiquitin-conjugated-specific HRP-linked antibody (Enzo Life Sciences).

Overexpression of Cish in HEK293 Cells

HEK293 cells (from ATCC) were transfected with pcDNA 3.1 mammalian expression vector (pcDNA)-Cish or pcDNA 3.1 (as control) using the FuGENE HD reagent (Promega) as detailed in the Supplemental Information.

Human 30-Plex Cytokine Assay

Primary human macrophages were infected with *Mtb* H37Rv at a MOI of 1 for 5 or 24 hr; cell culture supernatants were then filtered using a 0.22 μm PVDF filter, sampled, and stored at −80°C until analysis. GM-CSF release was quantified using the Cytokine Human Magnetic 30-Plex kit (Life Technologies) according to the manufacturer's protocol.

Statistical Analysis

In this work, statistical analyses were performed using Student's t test, with the exceptions of Figures 3B and 3F, for which the Wilcoxon Mann-Whitney test was used.

In Vivo Experiments

Animal studies were carried out in strict accordance with the recommendations from the Animal Protection Law in Korea. The protocol was approved by the Institutional Animal Care and Use Committee of Institut Pasteur Korea. All efforts were made to minimize suffering of the animals. 6-week-old female C57BL/6 Cish KO (previously described by Matsumoto et al., 1999) and WT mice (ORIENTBIO, South Korea) were challenged with *Mtb* H37Rv. More details on these experiments are described in the Supplemental Information.

Human Sample Analysis

Monocytes were purified from blood samples obtained from healthy blood adult donors (age between 18 and 60 years) under strict anonymity (Etablissement Français du Sang "Nord de France," EFS, Lille). Blood samples were provided without a gender specification. The use of human samples was approved by the French Ministry of Education and Research under the agreement DC 2015-2575.

SUPPLEMENTAL INFORMATION

Supplemental Information includes Supplemental Experimental Procedures, seven figures, one table, and one movie and can be found with this article online at http://dx.doi.org/10.1016/j.celrep.2017.08.101.

AUTHOR CONTRIBUTIONS

Conceptualization, C.J.Q., J.-P.C., A.Y., E.Y., and P.B.; Methodology, C.J.Q., J.-P.C., J.-M.S., S.-J.P., and S.T.; Software, C.J.Q., O.-R.S., G.D., and V.D.; Formal Analysis, C.J.Q., A.B., J.-M.S., and N.D.; Investigation, C.J.Q., O.-R.S., J.-P.C., and P.B.; Writing – Original Draft, C.J.Q., J.-P.C., R.B., E.Y., and P.B.; Writing – Review & Editing, C.J.Q., R.B., E.Y., and P.B.; Funding Acquisition, P.B. and R.B.; Resources, J.-M.S., N.D., S.J., R.I., A.-S.D., S.-J.P., J.C.G., S.T., and A.Y.; Supervision, P.B.

ACKNOWLEDGMENTS

We gratefully acknowledge T. Christophe, D. Fenistein, F. Ewann, and F. Lafont for technical assistance and C. Locht and A. Baulard for critical advices. Financial support for this work was provided by the European Union (ERC-STG INTRACELLTB 260901, MM4TB 260872, CycloNHit 608407, and TBVAC2020 643381), the ANR (ANR-10-EQPX-04-01, ANR-11-LABX-0024, ANR-14-CE14-0024, and ANR-14-CE08-0017), the Institut Pasteur (PTR441 and PTR22-16), the Feder (12001407 (D-AL) Equipex Imaginex BioMed), and the Région Nord Pas de Calais (12000080).

Received: April 28, 2017
Revised: July 29, 2017
Accepted: August 30, 2017
Published: September 26, 2017

REFERENCES

Armstrong, J.A., and Hart, P.D. (1971). Response of cultured macrophages to *Mycobacterium tuberculosis,* with observations on fusion of lysosomes with phagosomes. J. Exp. Med. *134*, 713–740.

Augenstreich, J., Arbues, A., Simeone, R., Haanappel, E., Wegener, A., Sayes, F., Le Chevalier, F., Chalut, C., Malaga, W., Guilhot, C., et al. (2017). ESX-1 and phthiocerol dimycocerosates of *Mycobacterium tuberculosis* act in concert to cause phagosomal rupture and host cell apoptosis. Cell. Microbiol. *19*, e12726.

Bhardwaj, S., Srivastava, N., Sudan, R., and Saha, B. (2010). Leishmania interferes with host cell signaling to devise a survival strategy. J. Biomed. Biotechnol. *2010*, 109189.

Bolte, S., and Cordelières, F.P. (2006). A guided tour into subcellular colocalization analysis in light microscopy. J. Microsc. *224*, 213–232.

Brodin, P., Poquet, Y., Levillain, F., Peguillet, I., Larrouy-Maumus, G., Gilleron, M., Ewann, F., Christophe, T., Fenistein, D., Jang, J., et al. (2010). High content phenotypic cell-based visual screen identifies *Mycobacterium tuberculosis* acyltrehalose-containing glycolipids involved in phagosome remodeling. PLoS Pathog. *6*, e1001100.

Carralot, J.P., Kim, T.K., Lenseigne, B., Boese, A.S., Sommer, P., Genovesio, A., and Brodin, P. (2009). Automated high-throughput siRNA transfection in raw 264.7 macrophages: a case study for optimization procedure. J. Biomol. Screen. *14*, 151–160.

Christophe, T., Jackson, M., Jeon, H.K., Fenistein, D., Contreras-Dominguez, M., Kim, J., Genovesio, A., Carralot, J.P., Ewann, F., Kim, E.H., et al. (2009). High content screening identifies decaprenyl-phosphoribose 2′ epimerase as a target for intracellular antimycobacterial inhibitors. PLoS Pathog. *5*, e1000645.

Flannagan, R.S., Cosío, G., and Grinstein, S. (2009). Antimicrobial mechanisms of phagocytes and bacterial evasion strategies. Nat. Rev. Microbiol. *7*, 355–366.

Foster, J.W. (2004). Escherichia coli acid resistance: tales of an amateur acidophile. Nat. Rev. Microbiol. *2*, 898–907.

Hamon, M., Bierne, H., and Cossart, P. (2006). Listeria monocytogenes: a multifaceted model. Nat. Rev. Microbiol. *4*, 423–434.

Kamura, T., Maenaka, K., Kotoshiba, S., Matsumoto, M., Kohda, D., Conaway, R.C., Conaway, J.W., and Nakayama, K.I. (2004). VHL-box and SOCS-box domains determine binding specificity for Cul2-Rbx1 and Cul5-Rbx2 modules of ubiquitin ligases. Genes Dev. *18*, 3055–3065.

Kaufmann, S.H. (2008). Immunology's foundation: the 100-year anniversary of the Nobel Prize to Paul Ehrlich and Elie Metchnikoff. Nat. Immunol. *9*, 705–712.

Khor, C.C., Vannberg, F.O., Chapman, S.J., Guo, H., Wong, S.H., Walley, A.J., Vukcevic, D., Rautanen, A., Mills, T.C., Chang, K.-C., et al. (2010). CISH and susceptibility to infectious diseases. N. Engl. J. Med. *362*, 2092–2101.

Koo, M.-S., Subbian, S., and Kaplan, G. (2012). Strain specific transcriptional response in *Mycobacterium tuberculosis* infected macrophages. Cell Commun. Signal. *10*, 2.

Landsman, T., and Waxman, D.J. (2005). Role of the cytokine-induced SH2 domain-containing protein CIS in growth hormone receptor internalization. J. Biol. Chem. *280*, 37471–37480.

Lehtonen, A., Matikainen, S., Miettinen, M., and Julkunen, I. (2002). Granulocyte-macrophage colony-stimulating factor (GM-CSF)-induced STAT5 activation and target-gene expression during human monocyte/macrophage differentiation. J. Leukoc. Biol. *71*, 511–519.

Majlessi, L., and Brosch, R. (2015). *Mycobacterium tuberculosis* meets the cytosol: the role of cGAS in anti-mycobacterial immunity. Cell Host Microbe *17*, 733–735.

Masuhara, M., Sakamoto, H., Matsumoto, A., Suzuki, R., Yasukawa, H., Mitsui, K., Wakioka, T., Tanimura, S., Sasaki, A., Misawa, H., et al. (1997). Cloning and characterization of novel CIS family genes. Biochem. Biophys. Res. Commun. *239*, 439–446.

Matsumoto, A., Seki, Y., Kubo, M., Ohtsuka, S., Suzuki, A., Hayashi, I., Tsuji, K., Nakahata, T., Okabe, M., Yamada, S., and Yoshimura, A. (1999). Suppression of STAT5 functions in liver, mammary glands, and T cells in cytokine-inducible SH2-containing protein 1 transgenic mice. Mol. Cell. Biol. *19*, 6396–6407.

McDonough, J.A., Newton, H.J., Klum, S., Swiss, R., Agaisse, H., and Roy, C.R. (2013). Host pathways important for *Coxiella burnetii* infection revealed by genome-wide RNA interference screening. MBio *4*, e00606–e00612.

Pethe, K., Swenson, D.L., Alonso, S., Anderson, J., Wang, C., and Russell, D.G. (2004). Isolation of *Mycobacterium tuberculosis* mutants defective in the arrest of phagosome maturation. Proc. Natl. Acad. Sci. USA *101*, 13642–13647.

Philips, J.A., and Ernst, J.D. (2012). Tuberculosis pathogenesis and immunity. Annu. Rev. Pathol. *7*, 353–384.

Piessevaux, J., De Ceuninck, L., Catteeuw, D., Peelman, F., and Tavernier, J. (2008). Elongin B/C recruitment regulates substrate binding by CIS. J. Biol. Chem. *283*, 21334–21346.

Queval, C.J., Song, O.R., Delorme, V., Iantomasi, R., Veyron-Churlet, R., Deboosère, N., Landry, V., Baulard, A., and Brodin, P. (2014). A microscopic phenotypic assay for the quantification of intracellular mycobacteria adapted for high-throughput/high-content screening. J. Vis. Exp. *83*, e51114.

Queval, C.J., Song, O.R., Deboosère, N., Delorme, V., Debrie, A.S., Iantomasi, R., Veyron-Churlet, R., Jouny, S., Redhage, K., Deloison, G., et al. (2016). STAT3 represses nitric oxide synthesis in human macrophages upon *Mycobacterium tuberculosis* infection. Sci. Rep. *6*, 29297.

Rascle, A., and Lees, E. (2003). Chromatin acetylation and remodeling at the Cis promoter during STAT5-induced transcription. Nucleic Acids Res. *31*, 6882–6890.

Russell, D.G. (2001). *Mycobacterium tuberculosis:* here today, and here tomorrow. Nat. Rev. Mol. Cell Biol. *2*, 569–577.

Simeone, R., Bobard, A., Lippmann, J., Bitter, W., Majlessi, L., Brosch, R., and Enninga, J. (2012). Phagosomal rupture by *Mycobacterium tuberculosis* results in toxicity and host cell death. PLoS Pathog. *8*, e1002507.

Simeone, R., Sayes, F., Song, O., Gröschel, M.I., Brodin, P., Brosch, R., and Majlessi, L. (2015). Cytosolic access of *Mycobacterium tuberculosis:* critical impact of phagosomal acidification control and demonstration of occurrence in vivo. PLoS Pathog. *11*, e1004650.

Stanley, S.A., Johndrow, J.E., Manzanillo, P., and Cox, J.S. (2007). The type I IFN response to infection with *Mycobacterium tuberculosis* requires ESX-1-mediated secretion and contributes to pathogenesis. J. Immunol. *178*, 3143–3152.

Sturgill-Koszycki, S., Schlesinger, P.H., Chakraborty, P., Haddix, P.L., Collins, H.L., Fok, A.K., Allen, R.D., Gluck, S.L., Heuser, J., and Russell, D.G. (1994). Lack of acidification in *Mycobacterium* phagosomes produced by exclusion of the vesicular proton-ATPase. Science *263*, 678–681.

Sun-Wada, G.H., Tabata, H., Kawamura, N., Aoyama, M., and Wada, Y. (2009). Direct recruitment of H+-ATPase from lysosomes for phagosomal acidification. J. Cell Sci. *122*, 2504–2513.

van der Wel, N., Hava, D., Houben, D., Fluitsma, D., van Zon, M., Pierson, J., Brenner, M., and Peters, P.J. (2007). *M. tuberculosis* and *M. leprae* translocate from the phagolysosome to the cytosol in myeloid cells. Cell *129*, 1287–1298.

Verdier, F., Chrétien, S., Muller, O., Varlet, P., Yoshimura, A., Gisselbrecht, S., Lacombe, C., and Mayeux, P. (1998). Proteasomes regulate erythropoietin receptor and signal transducer and activator of transcription 5 (STAT5) activation. Possible involvement of the ubiquitinated Cis protein. J. Biol. Chem. *273*, 28185–28190.

Vieira, O.V., Botelho, R.J., and Grinstein, S. (2002). Phagosome maturation: aging gracefully. Biochem. J. *366*, 689–704.

Wang, J., Teng, J.L., Zhao, D., Ge, P., Li, B., Woo, P.C., and Liu, C.H. (2016). The ubiquitin ligase TRIM27 functions as a host restriction factor antagonized by *Mycobacterium tuberculosis* PtpA during mycobacterial infection. Sci. Rep. *6*, 34827.

Wong, D., Bach, H., Sun, J., Hmama, Z., and Av-Gay, Y. (2011). *Mycobacterium tuberculosis* protein tyrosine phosphatase (PtpA) excludes host vacuolar-H+-ATPase to inhibit phagosome acidification. Proc. Natl. Acad. Sci. USA *108*, 19371–19376.

Yoshimura, A., Ohkubo, T., Kiguchi, T., Jenkins, N.A., Gilbert, D.J., Copeland, N.G., Hara, T., and Miyajima, A. (1995). A novel cytokine-inducible gene CIS encodes an SH2-containing protein that binds to tyrosine-phosphorylated interleukin 3 and erythropoietin receptors. EMBO J. *14*, 2816–2826.

Yoshimura, A., Naka, T., and Kubo, M. (2007). SOCS proteins, cytokine signalling and immune regulation. Nat. Rev. Immunol. *7*, 454–465.

Yoshimura, A., Suzuki, M., Sakaguchi, R., Hanada, T., and Yasukawa, H. (2012). SOCS, inflammation, and autoimmunity. Front. Immunol. *3*, 20.

Zhang, J.G., Farley, A., Nicholson, S.E., Willson, T.A., Zugaro, L.M., Simpson, R.J., Moritz, R.L., Cary, D., Richardson, R., Hausmann, G., et al. (1999). The conserved SOCS box motif in suppressors of cytokine signaling binds to elongins B and C and may couple bound proteins to proteasomal degradation. Proc. Natl. Acad. Sci. USA *96*, 2071–2076.

Zhang, W., Cheng, G.Z., Gong, J., Hermanto, U., Zong, C.S., Chan, J., Cheng, J.Q., and Wang, L.H. (2008). RACK1 and CIS mediate the degradation of BimEL in cancer cells. J. Biol. Chem. *283*, 16416–16426.

Zhao, J., Beyrakhova, K., Liu, Y., Alvarez, C.P., Bueler, S.A., Xu, L., Xu, C., Boniecki, M.T., Kanelis, V., Luo, Z.-Q., et al. (2017). Molecular basis for the binding and modulation of V-ATPase by a bacterial effector protein. PLoS Pathog. *13*, e1006394.

Cell Reports, Volume *20*

Supplemental Information

Mycobacterium tuberculosis Controls Phagosomal Acidification by Targeting CISH-Mediated Signaling

Christophe J. Queval, Ok-Ryul Song, Jean-Philippe Carralot, Jean-Michel Saliou, Antonino Bongiovanni, Gaspard Deloison, Nathalie Deboosère, Samuel Jouny, Raffaella Iantomasi, Vincent Delorme, Anne-Sophie Debrie, Sei-Jin Park, Joana Costa Gouveia, Stanislas Tomavo, Roland Brosch, Akihiko Yoshimura, Edouard Yeramian, and Priscille Brodin

Supplemental Information

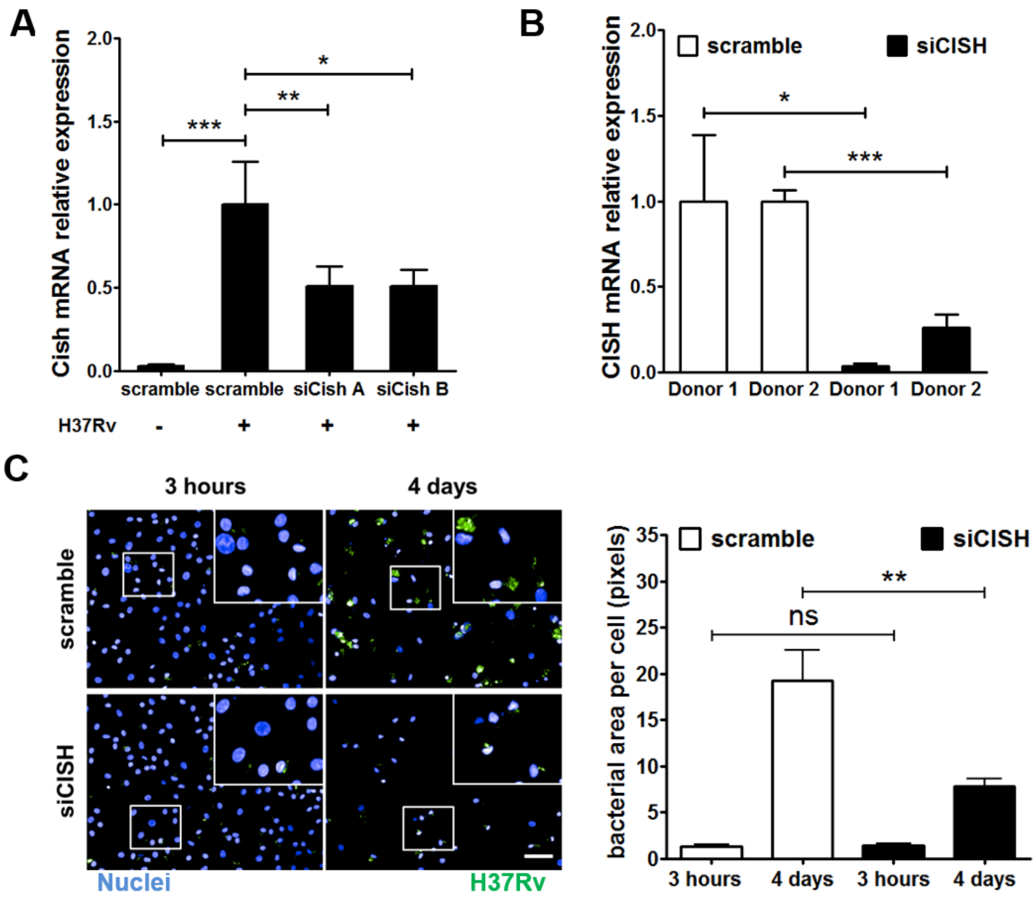

Supplemental Figure S1, Related to Figure 1. Inhibition of *Mtb* intracellular replication by CISH silencing

(**A**) Cish mRNA expression by RT-qPCR in siCishA, siCishB or scramble RAW 264.7 cells upon infection with H37Rv at 3 h p.i. (**B**) CISH mRNA relative expression quantified by RT-qPCR in siCISH and scramble hMΦ upon infection. The data reported correspond to the CISH mRNA expression ±SD obtained for two independent donors (black bars: donor 1; grey bar: donor 2) (**C**) Typical confocal images of siCISH and scramble hMΦ upon infection with H37Rv-GFP. Image based quantification of the infection 4 days p.i. For all data, statistical significances were determined using student t-test. * p<0.05, ** p<0.01, *** p<0.001, NS: not significant.

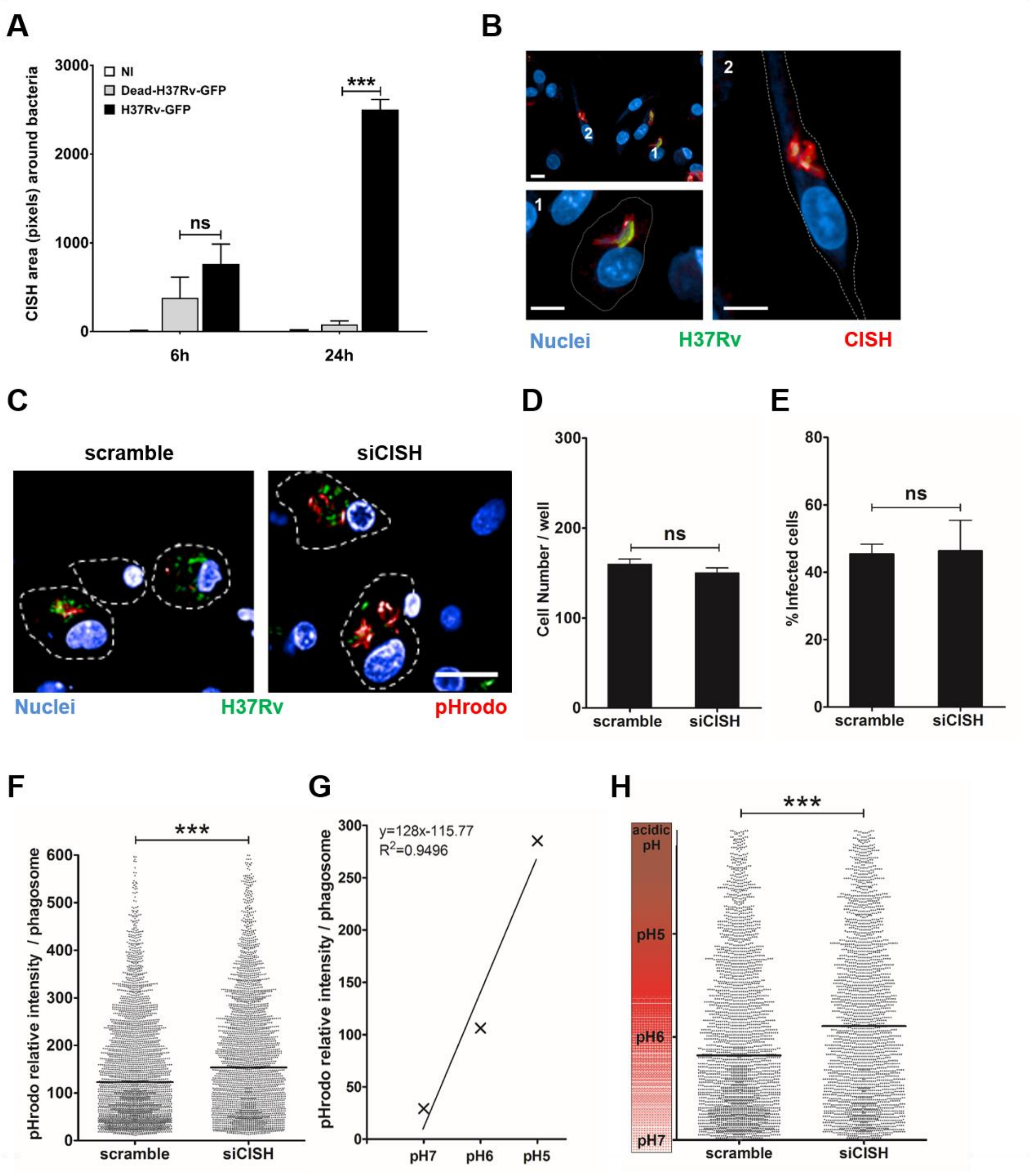

Supplemental Figure S2, Related to Figure 2. Vacuolar recruitment of CISH and phagosomal acidification upon *Mtb* infection in human primary macrophages (**A**) Analysis of the recruitment of Cish around Mtb H37Rv and Formalin-killed H37Rv-GFP (Dead-H37Rv-GFP) fluorescence microscopy in RAW 264.7 cells. Plot represents the recruitment of CISH (area) detected around dead or live H37Rv-GFP bacteria. *** p

<0.001, ns: non-significant. (**B**) Intracellular localization of CISH, in hMΦ infected with H37Rv-GFP for 6 h, was visualized using fluorescence microscopy. Nuclei were labelled with DAPI (blue), H37Rv-GFP bacteria were visualized in green and CISH was immunolabelled using CISH antibody (red). Scale bar, 10 µm. During the image acquisition an intensity cut-off was applied to avoid fluorescence saturation, which implies that lower concentrations of CISH localized elsewhere in the cells might not be visible in these images (**C**-**H**) Comparison of *Mtb*-containing phagome acidification in hMΦ, control (scramble) or silenced for CISH (siCISH), and infected at MOI 2 for 24 h with H37Rv-GFP labelled with pHrodo dye. (**C**) Typical confocal microscope images of hMΦ infected with pHrodo-labelled H37Rv-GFP Scale bar, 20 µm. (**D** and **E**) Image-based quantification of cell number (**D**) and percentage of infected cells (**E**). (**F**) Mean pHrodo relative intensity per phagosome. Plotted values are for siCISH (N phagosomes = 2860) and scramble controls (N phagosomes = 2920). Black bars represent the medians. (**G**) Standard curve representing the pHrodo relative intensity of bacteria in function of pH (**H**) Estimation of phagosomal pH obtained by converting pHrodo relative intensity / phagosome (**F**) in pH value according to the standard curve. Black bars represent the medians. All data are representative of 2 independent experiments, each performed with 4 replicates. ns: no-significant, ***p<0.001 (Mann-Whitney test).

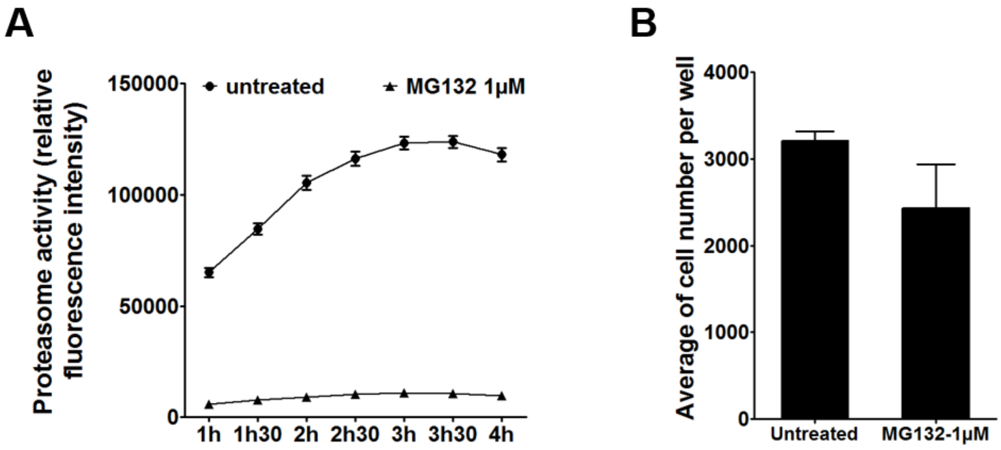

Supplemental Figure S3, Related to Figure 3. Monitoring proteasome activity in *Mtb*-infected macrophages (A) Kinetics of proteasome 20S activity in hMΦ treated with proteasome inhibitor MG132 (1 µM) or not (Untreated). **(B)** Control of cell viability by assessment of the number of cells in presence of MG132.

A

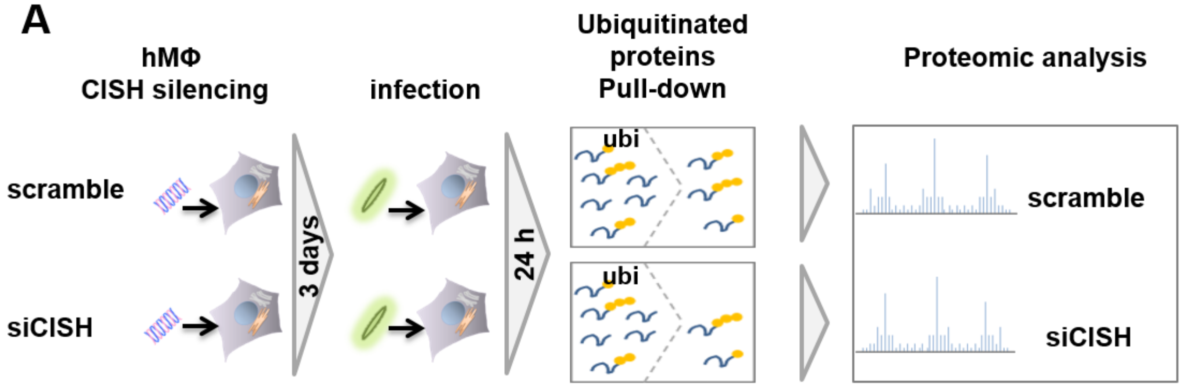

B

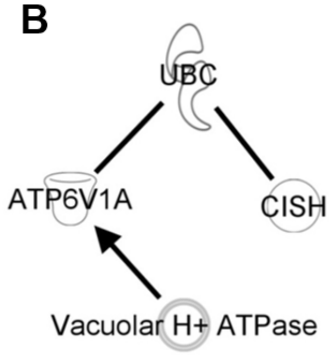

C

		Number of peptides			Number of spectra		
Protein name	accession number	siCISH	scramble	ratio	siCISH	scramble	ratio
ATP6V1A	P38606	6 ±1.4	10 ±1.4	0.6	7.5 ±0.7	15 ±1.4	0.5

Supplemental Figure S4, Related to Figure 4. High throughput proteomic analysis for the identification of V-ATPase subunit A as a target of CISH for ubiquitylation (**A**) Pull-down of ubiquitylated-proteins and their analysis by high throughput proteomics of extracts of siCISH and scramble hMΦ after *Mtb* H37Rv-GFP infection. (**B**) Scheme displaying the interactions between CISH, the ubiquitin system (UBC) and VATPase subunit A (ATP6V1A). (**C**) Number of peptides and spectra detected by proteomics for the identification of ATP6V1A as a potential CISH target for ubiquitination. Values represent the mean±SD of the peptides and spectra detected in *Mtb*-infected siCISH or scramble hMφ from two donors. The complete data are presented in Table S1.

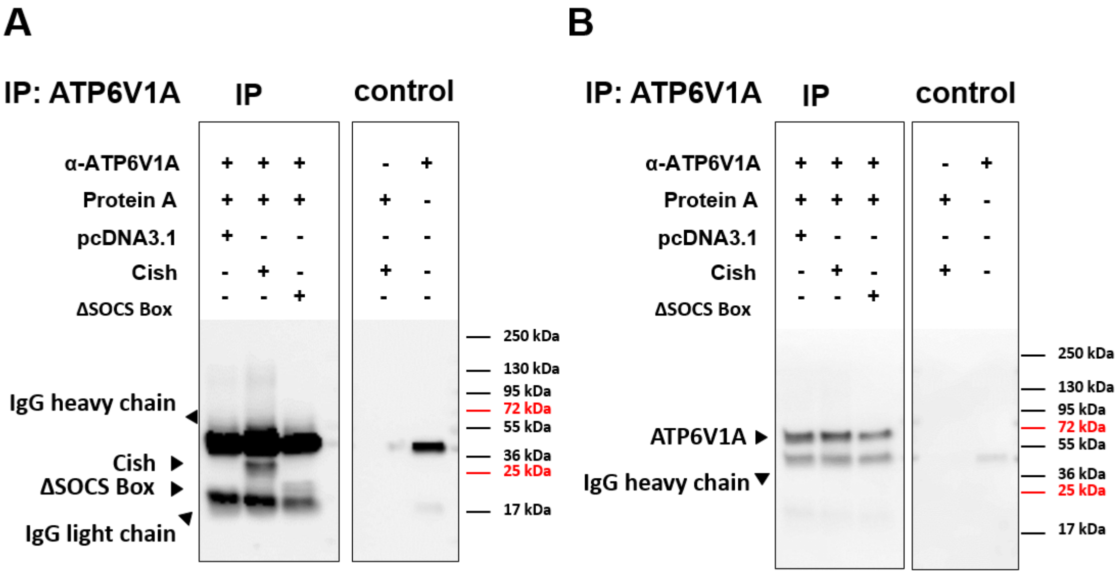

Supplemental Figure S5, Related to Figure 4. Cish interacts with V-ATPase subunit A (**A** and **B**) Whole immunoblot obtained after immunoprecipitation of endogenous ATP6V1A in HEK 293 cells transfected with pcDNA 3.1-Cish, pcDNA 3.1-CishΔSOCS Box or control empty vector pcDNA 3.1. (**A**) Immunoblotting of Cish showing the presence of Cish in cells transfected with pcDNA 3.1-Cish with contamination of immunoglobulin used for immunoprecipitation (heavy and light chain). (**B**) Membrane was then stripped and probed using an anti-ATP6V1A.

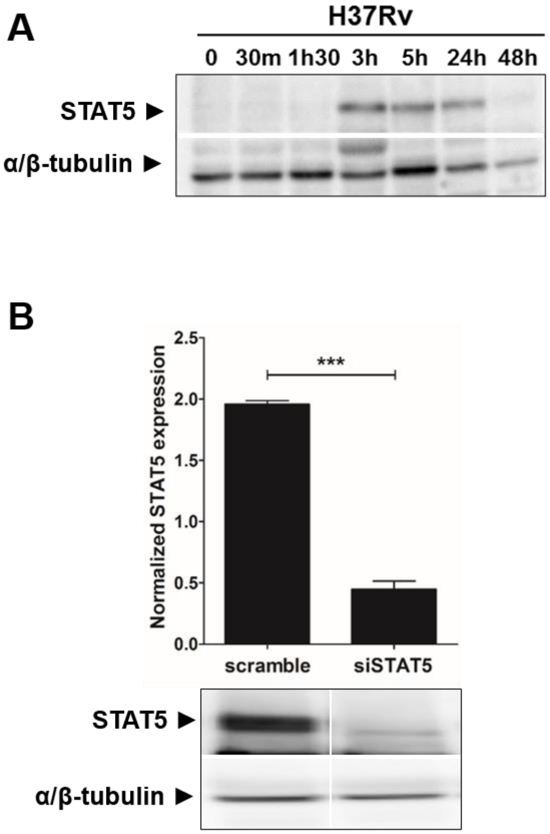

Supplemental Figure S6, Related to Figure 5. STAT5 activation in *Mtb*-infected macrophages is required for CISH expression (**A**) Kinetics of STAT5 activation in RAW 264.7 macrophages infected with H37Rv. STAT5 activation was analyzed by immunoblotting using anti-PY^{694}-STAT5. Blotting with anti-α/β-tubulin was used to confirm gel loading. Immunoblots are representative of two independent experiments. (**B)** siSTAT5 and scramble hMΦ were infected with H37Rv-GFP for 4 h. The graphic represents the quantification of STAT5 relative phosphorylation. Reported values represent the means ±SEM of three independent experiments. For all data, statistical significances were determined using student t-test. * $p<0.05$, ** $p<0.01$, *** $p<0.001$, ns: not significant.

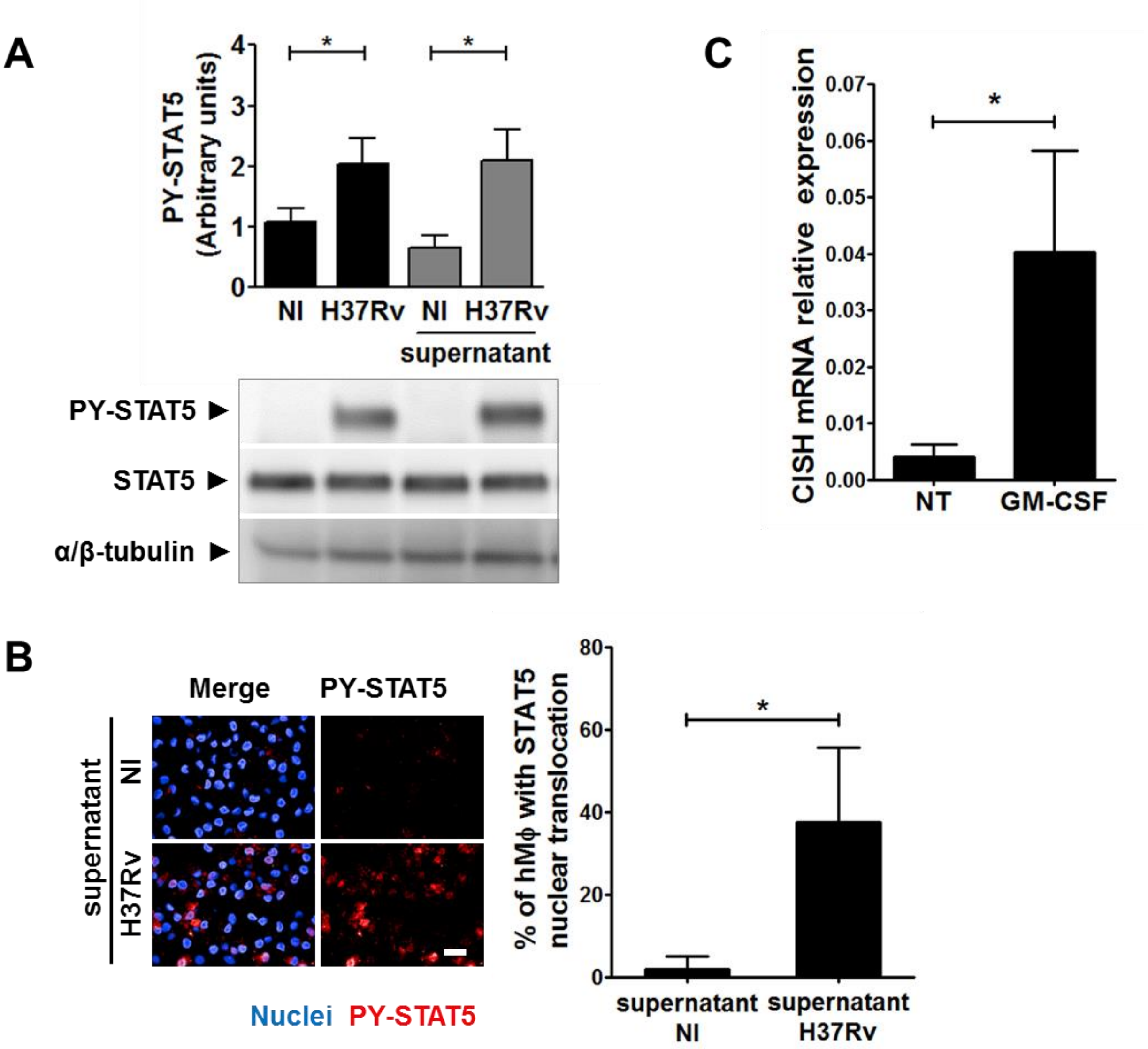

Supplemental Figure S7, Related to Figure 6. STAT5-mediated CISH expression depends on GM-CSF release by *Mtb*-infected macrophages. (**A)** STAT5 activation was analyzed by immunoblotting using anti-PY^{694}-STAT5 in hMΦ that had been infected with *Mtb* (H37Rv, black box) or not (NI, black box) or incubated with cell culture supernatant from hMΦ (H37Rv, grey box) and non-infected hMΦ (NI, grey box). Probing with anti-STAT5 and anti-α/β-tubulin antibodies was used to confirm gel loading. (**B**) Confocal images of hMΦ that were incubated for 3 h with filtered supernatant from uninfected hMΦ (NI) or supernatant from infected hMΦ (H37Rv). Macrophage nuclei were stained using DAPI (blue) and phosphorylated form STAT5 were detected using anti-PY^{694}-STAT5 (red). Micrographs are representative of four independent

experiments with four different donors. Scale bar, 20 µm. The graphic represents the mean percentage of hMΦ presenting STAT5 nuclear translocation. (**C**) Monitoring of CISH mRNA expression by RT-qPCR in hMΦ that were treated with 50 ng/mL of human GM-CSF for 3 h. Non-treated cells (NT) were used as negative control. The data correspond to the means ±SD of relative CISH mRNA expression from 2 independent experiments. For all data, statistical significances were determined using student t-test. * $p<0.05$, ** $p<0.01$, *** $p<0.001$, ns: not significant.

Supplemental Experimental Procedures

Antibodies and reagents

Polyclonal rabbit anti-PY^{694}-STAT5 (#9359), anti-STAT5 (#9363), anti-CISH (#8731) and anti-α/β-tubulin (#2148) were purchased from Cell Signaling Technology. Mouse anti-Poly-Ub (FK1/#BML-PW8805) was from ENZO. Rabbit Anti-ATP6V1A antibody (#17115-1-AP) was purchased from ProteinTech. For western blotting, the antibodies were probed using secondary donkey anti-rabbit antibodies, conjugated with horseradish peroxidase (Jackson Immunoresearch). For immunofluorescence, the antibodies were probed using secondary donkey anti-rabbit antibodies conjugated to Rhodamine Red X (Jackson Immunoresearch). Cell nuclei were fluorescently labelled using DAPI (Sigma-Aldrich) or SYTO60 (Life Technologies). Purified human GM-CSF and pure grade neutralizing anti-GM-CSF antibody were purchased from Miltenyi Biotech.

SiRNA individual duplexes

Non-targeting siRNA (scramble, target sequence: 5'-U AAG GCU AUG AAG AGA UAC-3') from Dharmacon Thermofisher (Boulder, CO, USA), siCishA (5'-CAGTTATACAGTATTTATTTA-3'), siCishB (5’-AAAGCAAGTGTTAGAACACAA-3’) were resuspended at 2 µM in QIAGEN resuspension buffer, aliquoted and stored at -20°C until use.

Mammalian cells

RAW 264.7 cells (ATCC TIB-71) were grown to 60-80% confluence in RPMI 1640 medium (Difco) supplemented with 10% heat-inactivated FBS (Life Technologies). CD14^{+} hMΦs were isolated from human peripheral blood monocytes (hPBMCs) as previously described (Queval et al., 2014). Briefly, hPBMCs were purified by Ficoll density gradient centrifugation and isolated using a CD14^{+} magnetic bead purification kit (Miltenyi Biotech). Purified CD14^{+} monocytes were then differentiated into macrophages by 6-day incubation in RPMI 1640 complemented with 10% FBS and 40 ng/mL colony stimulating factor (hM-CSF) (Miltenyi Biotech) at 37°C in 5% CO_2. For all secondary cell lines used in this manuscript, the maximum number of passage after thawing was 10.

Mycoplasma test was routinely performed and all secondary cell lines used were tested negative for mycoplasma.

Mycobacterial strains and cell infection

A recombinant strain of *Mtb* H37Rv expressing the enhanced green fluorescent protein (H37Rv-GFP) was cultured in Middlebrook 7H9 medium (Difco) supplemented with 10% oleic acid-albumin-dextrose-catalase (OADC, Difco), 0.2% glycerol, 0.05% Tween 80 (all from Sigma-Aldrich, St. Louis, MO, USA), and 50 µg/ml hygromycin (Invitrogen) until the exponential phase was reached (Christophe et al., 2009). Bacilli were washed with Dulbecco's Phosphate Buffered Saline (DPBS free from $MgCl_2$ and $CaCl_2$, Gibco) resuspended in 10 mL of RPMI 1640 medium containing 10% FBS and decanted for 1 h at room temperature to allow bacterial aggregates to sediment. Bacterial titre was determined by measuring EGFP fluorescence on a Victor Multilabel Counter (Perkin Elmer). The bacterial suspension was diluted at the required titre in RPMI 1640 supplemented with 10% FBS prior to infection.

***In vivo* experiments**

6 week-old female C57BL/6 Cish knock-out mice (previously described elsewhere(Matsumoto et al., 1999)) and wild-type mice (ORIENTBIO Inc., SouthKorea) were challenged with *Mtb* H37Rv via the intranasal route with 10 µl of a suspension containing 5.10^5 organisms/ml. The day of infection (day 0 (n=4), 21 (n=4) and 42 days (n=4), lungs were homogenized by use of an MM300 apparatus (Qiagen) and 2.5-mm diameter glass beads. Serial 10-fold dilutions in medium were plated on 7H11 agar and colony forming unit counts were ascertained at 37°C after 3 weeks of growth. CFUs are reported for 4 mice per group per time point. Each cage hosted different groups. The experimentator had access to the label of the cages and, for each group, took mice randomly in the cages for each time point.

CFU determination

Cells were washed before lysis at the indicated times after bacterial infection. Serial dilutions were performed in DPBS and three drops of each dilution were plated onto

7H11 agar plates supplemented with 10% OADC. *Mtb* colony numbers were determined and the average of three spots of at least two dilutions were considered to determine the intracellular CFU per well. The CFUs were normalized to the total cell numbers (CFU/well) of the corresponding wells, using data from image analysis.

Cell-based siRNA assays

RAW 264.7 cells were reverse transfected with 100 nM of siRNA, in duplicate, using 0.2% Lipofectamine LTX (Invitrogen) in 384-well tissue culture plates (Greiner, Frickenhausen, Germany). Non-targeting siRNA (scramble) from Dharmacon Thermofisher (Boulder, CO, USA) was used to transfect cells in 48 wells per 384-well assay plate. Three days after transfection, one replicate of transfected cells was infected with H37Rv-GFP at a multiplicity of infection (MOI) of 3. Infection was allowed to proceed for 5 h at 37°C and 5% CO_2, and extracellular bacilli were removed via extensive washing of the plate, five times with 60 µl of DPBS-1% FBS using the Aquamax DW4 plate washer from Molecular Devices (Sunnyvale, CA, USA). Antibiotics were never used at any of the multiple steps of the experiments. Infected RAW 264.7 cells were resuspended in 50 µl of RPMI with 10% FBS and incubated at 37°C and 5% CO_2 for 5 days. As a positive control, 32 wells per 384-well plate were treated with 5 µg/ml of isoniazid (INH) from Sigma-Aldrich. On day 5 after infection, cells were stained with 10 µM of SYTO60 far red fluorescent nucleic acid stain (Invitrogen) and confocal images were acquired using an automated fluorescence microscope Opera (Perkin Elmer) equipped with a 20×/0.7 NA water immersion lens, driven by a robotic platform from EVOTEC AG (Hamburg, Germany). Of the four available light sources, 488- and 635-nm confocal excitation lines were used in order to excite GFP and SYTO60 red, respectively. Emission was detected simultaneously using two CCD cameras in spectral bands at 535 and 690 nm. Three fields for each well were acquired. Images from 384-well plates were analyzed as described previously (Christophe et al., 2009). To detect the intracellular bacteria, each field was analyzed using image-based analysis software Acapella 2.6 (Perkin Elmer). For each field, two channels were recorded: one for the green fluorescent-bacteria (green) and one for the cell nuclei (blue channel). 2-color images were then segmented using the following implementation of procedures: *i*) nuclei

detection was performed using a built-in Acapella procedure, *ii*) cytoplasm detection, based on the nuclei population, was performed using a built-in Acapella procedure, *iii*) bacteria detection was performed by retaining only pixels whose intensities were higher than a manually-defined threshold, *iv*) to identify infected cells the positions of cells and those of bacteria were merged together. Final results, expressed as the averages of the corresponding fields, concerned, respectively, the total bacterial area, the total number of cells, the percentage of infected cells and the bacterial area per cell (average of all infected cells).

SiRNA transfection of human primary macrophages

SiRNA transfection of primary hMΦs was adapted from the protocol previously described (Troegeler et al., 2014). Briefly, 5 days after differentiation with human M-CSF (40 ng/mL), primary $CD14^+$ hMΦs were collected for reverse transfection using the HiPerFect transfection reagent from Qiagen. Silencing was performed with siRNA from Dharmacon. For STAT5 silencing, 50 nM of pooled STAT5A siRNA (D-005169-01, D-005169-02, D-005169-05, D-005169-06) and pooled STAT5B siRNA (D-010539-01, D-010539-03, D-010539-05, D-010539-18) was used. For CISH silencing, 50 nM of pooled CISH siRNA (D-017381-01, D-017381-02, D-017381-03, D-017381-04) was used. Of note, in mice only one variant of Cish mRNA is expressed (NM_009895.3), whereas in humans 2 variants of CISH mRNAs are found (CISH variant 1: NM_013324.5; CISH variant 2: NM_145071.2). These pooled CISH siRNA target the two variants. As a control, macrophages were transfected with 50 nM of pooled Non-targeting siRNA (scramble) (D-001810-10). Transfected cells were then incubated for 3 days in RPMI 1640 supplemented with 10% FBS and 20 ng/mL M-CSF at 37°C, 5% CO_2. Transfected cells were then infected with *Mtb,* and samples were collected at the indicated time points.

Detection of CISH mRNA by RT-qPCR

After infection, macrophages were washed once with DPBS and mRNA was purified by using the RNeasy purification kit from QIAGEN, according to the manufacturer's recommendations. For each sample tested, 1 µg of mRNA was then reverse-transcribed

using the Reverse Transcription System kit from PROMEGA. Relative expression of *CISH* cDNA was detected using specific FAM-labeled Taqman Probe from Life Technologies which targeted mouse *Cish* (Mm01230623) or human *CISH* variants 1 and 2 (Hs00367082). We used *GAPDH* as a housekeeping gene. GAPDH was detected using FAM-labeled Taqman Probe from Life technologies targeting mouse *Gapdh* (Mm99999915) or human *GAPDH* (Hs02758991). FAM fluorescence was detected using LightCycler 480 (ROCHE), and the crossing points (Cp) of PCR reactions were calculated using the Basic Relative Quantification method. Data were then normalized and quantified using the $2^{-\Delta Cp}$ method.

Electrophoresis and immunoblotting

Cells were washed with DPBS (Difco), lysed using 62.5 mM Tris-HCl (pH 6.8), 10% glycerol, 4% SDS, 0.025% bromophenol blue and 1.5% β-mercaptoethanol, and boiled at 100°C for 15 min. The proteins were resolved by SDS-PAGE in precast acrylamide gels 4%-15% (BioRad) and transferred onto a polyvinylidene difluoride (PVDF) membrane. The PVDF membranes were blocked in DPBS, 0.1% Tween 20, 5% BSA and probed overnight with the appropriate antibodies (CISH 1/100, PY-STAT5 1/1000). Blots were then incubated with horseradish peroxidase-conjugated secondary antibodies (Jackson Immunoresearch), followed by detection using chemiluminescence, according to the manufacturer's instructions (Immobilon, Millipore). The chemiluminescent signal was detected using the LAS 3000 Pro Bio Imaging Analyser. The fluorescence intensity of each spot was quantified using the ImageJ software.

Immunofluorescence for image based-quantification of CISH, poly-Ub, V-ATPase subunit A and PY-STAT5

After infection, hMΦs were fixed with 10% neutral buffered Formalin solution (HT5014, Sigma-Aldrich) for 30 min and permeabilized with cold methanol for 10 min at -20°C for STAT5 detection or with PBS-Triton X-100 0.2% for 4 min at RT for CISH, ubiquitin and V-ATPase subunit A detection. Cells were then incubated with blocking buffer (DPBS, 1% FBS) for 30 min prior to overnight incubation at 4°C with rabbit anti-CISH (1/100), rabbit anti-Poly-Ub (1/100) and rabbit anti-PY^{694}-STAT5 (1/100) antibodies. Cells were

then washed 3 times with DPBS and incubated with donkey anti-rabbit conjugated with Rhodamine Red X for 1 h at room temperature. Cells were washed again 3 times with DPBS and incubated for 10 min with 2.5 µg/mL DAPI in DPBS. The buffer was replaced by D-PBS containing 1% FBS. Plates were sealed and stored at 4°C. Confocal images were acquired on an automated fluorescent confocal microscope Opera (Perkin Elmer) using a 20X or 60X water lens (NA 0.7), 561 nm laser and 600 nm camera. 8 fields per well were recorded on average. Each image was processed using Columbus image analysis software (Perkin Elmer). For the quantification of the percentage of colocalization between *Mtb* H37Rv and CISH (Fig. 2C), GFP-bacteria spots were first detected using a built-in Acapella procedure as described elsewhere(Queval et al., 2016). The size of each spot was extended by 3 pixels on each direction leading to "the bacterial ring region". CISH fluorescence area was detected in the red channel by retaining only pixels which intensity was higher than a manually-defined threshold. The ratio of the "bacterial ring region" that was positive for CISH was then quantified. For the quantification of STAT nuclear translocations, PY-STAT5 staining was segmented using built-in scripts, and the ratio of cells positive for PY-STAT5 was determined for each well (Fig. 5)(Queval et al., 2016).

For the quantification of CISH/V-ATPase colocalization around *Mtb* vacuole, confocal images were acquired using a confocal microscope Zeiss LSM880 equipped with a Plan-Apochromat 63x/1,4 NA lens and laser 405, 488, 561, 633 nm. During the image acquisition an intensity cut-off was applied to avoid fluorescence saturation, which implies that lower concentrations of CISH localized elsewhere in the cells might not be visible in these images. Fluorescence intensities from *Mtb*-GFP, CISH and V-ATPase subunit A were analyzed using ImageJ and the plugin JACoP (Bolte and Cordelieres, 2006). The colocalization between CISH and V-ATPase subunit A was determined by the calculation of the Pearson's correlation coefficient in the area that encompasses the bacteria by an extra 1 µm (Bolte and Cordelieres, 2006).

Quantification of phagosomal acidification by LysoTracker

3000 RAW 264.7 cells were reverse transfected in 384-well plates (Carralot et al., 2009) with 100 nM of non-targeting (scramble) siRNA or with CISH-targeting siRNA duplexes

A & B using 0.2% Lipofectamine LTX (Invitrogen), and further incubated at 37°C and 5% CO_2 for four days. As a positive control, three days after transfection, cells were activated with recombinant mouse Interferon-gamma (IFNγ, R&D Systems) and Lipopolysaccharide (LPS, Sigma) at 0.1 Units/ml and 10 ng/ml, respectively. Four days after transfection, cells were infected with *Mtb* H37Rv at an MOI of 10:1. Infection was allowed to proceed for 2 h at 37°C and 5% CO_2, and extracellular bacilli were extensively washed out 5 times with 60 µl of DPBS-1% FBS. Infected RAW 264.7 cells were labelled with 1 µM of LysoTracker red DND-99 (Invitrogen) and 10 µg/ml of Hoechst 33342 (Sigma-Aldrich) for 1 h at 37°C and 5 % CO_2. Cells were washed four times with 60 µl of DPBS-1% FBS then fixed with 40 µl of 10% formalin solution for 10 min. Cells were subsequently washed twice with 60 µl of DPBS-1% FBS and stored in 1% FCS in PBS at 4°C until image acquisition. Confocal images were acquired using an Opera automated fluorescence microscope equipped with a 40×/0.7 NA water immersion lens. 405-, 488-, and 561-nm confocal excitation lines were used in order to excite Hoechst 33342, EGFP and LysoTracker red, respectively. Emission was detected simultaneously using two CCD cameras in spectral bands at 450, 520 and 600 nm. Four fields for each well were measured. Image analysis for the quantification of "the total acidic compartment surface proximal to cell nuclei" was performed using an algorithm described previously and Columbus software (Brodin et al., 2010).

Quantification of phagosomal acidification by pHrodo

Prior to cell infection, a two weeks old Mtb H37Rv-GFP culture was washed twice with DPBS buffer and then was incubated for 1 h at 37°C in 100 mM sodium bicarbonate, pH 8.5, containing 0.5 mM of amine-reactive pHrodo Red succinimidyl ester (Lifetechnologies). Labelled-bacteria were washed 3 times by centrifugation in DPBS buffer. hMΦs were infected with pHrodo-labelled and GFP-expressing Mtb at a MOI of 2. 24 h after infection, macrophage nuclei were labelled with Hoechst 33342 and confocal images were acquired on live cells using an OPERA automated fluorescence microscope equipped with a 60X water immersion lens (NA 0.7). 405-, 488-, and 561-nm confocal excitation lines were used in order to excite Hoechst 33342, EGFP and pHrodo red, respectively. Emission was detected simultaneously using two CCD cameras in

spectral bands at 450, 520 and 600 nm. For the quantification of pHrodo intensity around phagosomes, GFP-bacteria spots were first detected using a built-in Acapella procedure as described elsewhere (Queval et al., 2016). The size of each spot was extended by 4 pixels leading to “bacterial phagosome region”. For each phagosome, pHrodo fluorescence intensity was detected in the red channel and data were plotted as mean relative pHrodo intensity per phagosome.

Determination of the standard curve pHrodo relative intensity / bacteria = f(pH):

Mtb H37rv-GFP labelled with pHrodo were incubated in CO_2 independent medium (Gibco) at various pH (pH10 to pH4). The stained bacteria were then imaged using automated confocal microscopy and analysed using the same parameters as those used in samples. pHrodo mean intensity was measured for each bacterium detected (at least N=100) and the intensity was plotted as a median pHrodo intensity per bacterium (as shown in Supplementary Fig. S2F).

Quantification of proteasome activity

hMΦs transfected with siCISH or scramble were plated in a 384-well microplate and infected for 24 h with H37Rv-DsRed. As control, scramble cells were treated for 4 h with proteasome inhibitor MG132 1µM. Proteasome activity was quantified using Amplite^{TM} Fluorimetric Proteasome 20S Activity Assay Kit from AAT Bioquest. Cells were incubated 3 h with Proteasome LLVY-R110 substrate and Assay buffer according to the manufacturer’s recommendations and the fluorescence was collected using a plate reader Victor X3 (PerkinElmer), with filters Ex/Em = 490/525 nm.

Pull-down of ubiquitinated-proteins

hMΦs were first transfected with siCISH or scramble prior to infection with H37Rv-GFP at MOI of 1. 24 h post-infection, cells were washed once with PBS and lysed using buffer containing 20mM Tris HCl pH 7.5, 140mM NaCl, 1mM EDTA, 2% CHAPS (3-[((3-cholamidopropyl)-dimethylammonio]-1-propanesulfonate)), 10% glycerol, 10mM N-Ethylmaleimide. Cells were then collected and incubated in lysis buffer for 1 h at 4°C. Ubiquitinated-proteins were then pulled-down using UbiQapture kit from Enzolifesciences. For each sample, 700 µg of cell lysate was incubated with 80 µL of

UbiQapture-Q Matrix for 4.5 h at 4°C. UbiQapture-Q Matrix was then washed 5 times with PBS. Ubiquitinated-proteins that were bound to UbiQapture-Q Matrix were then eluted by boiling at 95°C for 15 min in 150µL PBS-containing SDS-PAGE buffer (62.5 mM Tris HCl pH 6.8, 4% SDS). UbiQapture-Q Matrix was then removed by short centrifugation. Eluted fractions that contain Ubiquitinated-proteins were collected into microtubes and stored at -20°C until analysis.

Proteomic analysis

After denaturation in a buffer 10 mM Tris-HCl pH 8 containing 5% SDS, 5% β-mercaptoethanol, 1 mM EDTA and 10% glycerol for 3 min, protein samples were fractionated on 10% acrylamide SDS-PAGE gel. The electrophoretic migration was stopped when the protein sample migrated at 1-cm into the separating gel. The gel was briefly stained with Coomassie Blue, and five bands containing the whole sample were cut. In-gel digestion of gel slices was performed as previously described (Miguet et al., 2009). An UltiMate 3000 RSLC nano System (Thermo Fisher Scientific) was used for separation of the protein digests. Peptides were automatically fractionated onto a C18 reversed phase column (75 µm×150 mm, 2 µm particle, PepMap100 RSLC column, Thermo Fisher Scientific, temperature 35 °C). Trapping was performed during 4 min at 5 µL/min with solvent A (98 % H2O, 2% ACN and 0.1 % FA). Elution was carried out using two solvents A (0,1 % FA in water) and B (0,1 % FA in ACN) at a flow rate of 300 nL/min. Gradient separation was 3 min at 5% B, 37 min from 5 % B to 30% B, 5 min to 80% B, and maintained at 80% for 5 min. The column was equilibrated for 10 min with 5% buffer B prior to the next sample analysis. The eluted peptides from the C18 column were analyzed by Q-Exactive instruments (Thermo Fisher Scientific). The electrospray voltage was 1.9 kV, and the capillary temperature was 275 °C. Full MS scans were acquired in the Orbitrap mass analyzer over m/z 300–1200 range with resolution 35,000 (m/z 200). The target value was 5.00E+05. Ten most intense peaks with charge state between 2 and 4 were fragmented in the HCD collision cell with normalized collision energy of 27%, and tandem mass spectrum was acquired in the Orbitrap mass analyzer with resolution 17,500 at m/z 200. The target value was 1.00E+05. The ion selection threshold was 5.0E+04 counts, and the maximum allowed ion accumulation times were

250 ms for full MS scans and 100 ms for tandem mass spectrum. Dynamic exclusion was set to 30 s.

Proteomic data analysis

Raw data collected during nanoLC-MS/MS analyses were processed and converted into *.mgf peak list format with Proteome Discoverer 1.4 (Thermo Fisher Scientific). MS/MS data were interpreted using search engine Mascot (version 2.4.0, Matrix Science, London, UK) installed on a local server. Searches were performed with a tolerance on mass measurement of 0.2 Da for precursor and 0.2 Da for fragment ions, against a composite target-decoy database (40488 total entries) built with Human Swiss-Prot database (TaxID=9606, 20 January 2015, 20198 entries) fused with the sequences of recombinant trypsin and a list of classical contaminants (46 entries). Cysteine carbamidomethylation, methionine oxidation, protein N-terminal acetylation, cysteine propionamidation and lysine ubiquitination (Gly-Gly) were searched as variable modifications. Up to three trypsin-missed cleavages were allowed. For each sample, peptides were filtered out according to the cut-off set for protein hits with 1 or more peptides longer than 7 residues, ion score > 30, identity score > 0 and a false positive identification rate less of than 0.5%.

We then selected the most representative proteins by removing all proteins displaying a mean number of peptides <3 in scramble control macrophages, in two independent biological replicates. The proteins targeted by CISH for ubiquitination were then determined by comparison of the relative number of proteins detected in siCISH macrophages to that of scramble control. The list of proteins presented in Table S1 was obtained according to the following criteria: i) ratio (number of peptides siCISH/number of peptides scramble) < 0.6; ii) ratio (number of spectra siCISH/number of spectra scramble) < 0.6; iii) number of peptides scramble - number of peptides siCISH ≥3.

Immunoprecipitation of V-ATPase subunit A

To immununoprecipitate endogenous V-ATPase catalytic subunit A (ATP6V1A), hMΦs were transfected with siCISH or scramble prior to infection with H37Rv-GFP for 24 h. To block protein degradations by proteasome, infected cells were treated with proteasome

inhibitor MG132 1µM 5 h. Cells were lysed in CHAPS buffer as described above (Pull-down of ubiquitinated-proteins, Materials and Methods). For each sample, 1 mg of cell lysate was incubated for 5 h at 4°C with 30 µL of Protein A Sepharose CL-4B beads (GE Healthcare) in presence of rabbit anti-ATP6V1A antibody (Proteintech Europe). The immune complexes were successively washed twice with buffer 1 (20mM Tris HCl, pH 7.5, 140mM NaCl, 1mM EDTA, 0.5% CHAPS, 10% Glycerol, 10mM N-Ethylmaleimide) and further washed 3 times with buffer 2 (20mM Tris HCl, pH 7.5, 280mM NaCl, 1mM EDTA, 10% Glycerol, 10mM N-Ethylmaleimide). The immune complexes were finally boiled for 15 minutes in loading buffer (62.5 mM Tris, pH 6.8, 20% Glycerol, 1.5% SDS, 0.025% bromophenol blue, 8% β-mercaptoethanol) before analysis by SDS-PAGE. After electrophoresis and transfer into PVDF membrane, ATP6V1A was immunoblotted using rabbit anti-ATP6V1A (Novus) and secondary antibody easy blot anti-rabbit IgG conjugated with HRP (GeneTex). Ubiquitin was revealed using Ubiquitin-conjugated specific HRP-linked antibody (Enzo Life Sciences).

Transfection of HEK 293 cells with pcDNA 3.1 encoding for Cish

HEK 293 cells (from ATCC) were grown to 80% confluence in RPMI 1640 medium (Difco) supplemented with 10% heat-inactivated FBS (Life Technologies). One day before plasmid transfection, 10^6 HEK 293 cells were harvested in a T-25 flask with 8 mL of complete growth medium. For each condition 8 µg of control pcDNA 3.1 or pcDNA 3.1-Cish were diluted in PBS to a final volume of 375 µL and mixed with 25 µL of FuGENE HD reagent (Promega). Transfectant-DNA complexes were then incubated 10 min at room temperature before incubation with the cells for 48 h at 37°C in an atmosphere containing 5% CO_2.

Human 30-Plex cytokine assay

Primary hMΦs were infected with *Mtb* H37Rv at a MOI of 1.for 5 or 24 h, cell culture supernatants were then filtered using a 0.22 µM PVDF filter, sampled and stored at -80°C until analysis. GM-CSF release was quantified using Human cytokine Magnetic 30-plex kit (Life Technologies) according to the manufacturer's protocol.

Supernatant transfer assay

hMΦs were first infected with *Mtb* H37Rv at a multiplicity of infection (MOI) of 2 for 3 h, then the supernatant was collected and filtered using a 0.22 µm PVDF filter to remove any residual bacteria. Non-infected hMΦs were further incubated with filtered supernatant for 3 h. Cells were collected, and STAT5 activation was determined by western blotting.

Neutralization of GM-CSF activity

Primary $CD14^+$ hMΦs were infected for 3 h with *Mtb* H37Rv at a MOI of 2, in presence of 10 µg/mL of neutralizing anti-GM-CSF (α-GM-CSF) antibody or control IgG. Phosphorylation of STAT5 was analyzed by electrophoresis and immunoblotting as described above.

Statistical analysis

In this work statistical analyses were performed using Student's t-test, with the exception of Figures 3B and 3F, for which Wilcoxon Mann-Whitney test was used.

Ethics statement.

Animal studies were carried out in strict accordance with the recommendations from the Animal Protection Law in Korea. The protocol IPK-10008 entitled New Target for anti-TB drug development was approved by the Institutional Animal Care and Use Committee of Institut Pasteur Korea. All efforts were made to minimize suffering of the animals. Each group (WT and Cish KO) was hosted in different cages. Human monocytes were purified from blood samples obtained from healthy blood adult donors under strict anonymity (Etablissement Français du Sang "Nord de France", EFS, Lille). Written informed consents were obtained from the donors under EFS contract n°NT/18/2016/200 with respect to Decree n°2007-1220 (articles L1243-4, R1243-61 and following) dated August 10th 2007 of the French Public Health Code (published in the Official Journal of the French Republic of August 14th 2007). The use of human samples was approved by the French Ministry of Education and Research under the agreement DC 2015-2575.

References (Supplemental dataset)

Bolte, S., and Cordelieres, F.P. (2006). A guided tour into subcellular colocalization analysis in light microscopy. Journal of microscopy *224*, 213-232.

Brodin, P., Poquet, Y., Levillain, F., Peguillet, I., Larrouy-Maumus, G., Gilleron, M., Ewann, F., Christophe, T., Fenistein, D., Jang, J.*, et al.* (2010). High content phenotypic cell-based visual screen identifies Mycobacterium tuberculosis acyltrehalose-containing glycolipids involved in phagosome remodeling. PLoS pathogens *6*, e1001100.

Carralot, J.P., Kim, T.K., Lenseigne, B., Boese, A.S., Sommer, P., Genovesio, A., and Brodin, P. (2009). Automated high-throughput siRNA transfection in raw 264.7 macrophages: a case study for optimization procedure. Journal of biomolecular screening *14*, 151-160.

Christophe, T., Jackson, M., Jeon, H.K., Fenistein, D., Contreras-Dominguez, M., Kim, J., Genovesio, A., Carralot, J.P., Ewann, F., Kim, E.H.*, et al.* (2009). High content screening identifies decaprenyl-phosphoribose 2' epimerase as a target for intracellular antimycobacterial inhibitors. PLoS pathogens *5*, e1000645.

Matsumoto, A., Seki, Y., Kubo, M., Ohtsuka, S., Suzuki, A., Hayashi, I., Tsuji, K., Nakahata, T., Okabe, M., Yamada, S.*, et al.* (1999). Suppression of STAT5 functions in liver, mammary glands, and T cells in cytokine-inducible SH2-containing protein 1 transgenic mice. Molecular and cellular biology *19*, 6396-6407.

Miguet, L., Bechade, G., Fornecker, L., Zink, E., Felden, C., Gervais, C., Herbrecht, R., Van Dorsselaer, A., Mauvieux, L., and Sanglier-Cianferani, S. (2009). Proteomic analysis of malignant B-cell derived microparticles reveals CD148 as a potentially useful antigenic biomarker for mantle cell lymphoma diagnosis. Journal of proteome research *8*, 3346-3354.

Queval, C.J., Song, O.R., Deboosere, N., Delorme, V., Debrie, A.S., Iantomasi, R., Veyron-Churlet, R., Jouny, S., Redhage, K., Deloison, G.*, et al.* (2016). STAT3 Represses Nitric Oxide Synthesis in Human Macrophages upon Mycobacterium tuberculosis Infection. Scientific reports *6*, 29297.

Queval, C.J., Song, O.R., Delorme, V., Iantomasi, R., Veyron-Churlet, R., Deboosere, N., Landry, V., Baulard, A., and Brodin, P. (2014). A microscopic phenotypic assay for the quantification of intracellular mycobacteria adapted for high-throughput/high-content screening. Journal of visualized experiments : JoVE, e51114.

Troegeler, A., Lastrucci, C., Duval, C., Tanne, A., Cougoule, C., Maridonneau-Parini, I., Neyrolles, O., and Lugo-Villarino, G. (2014). An efficient siRNA-mediated gene silencing in primary human monocytes, dendritic cells and macrophages. Immunology and cell biology.
